# KMT2D loss drives adeno-to-squamous transition and sensitizes TKI-resistant lung cancer to AURKA inhibition

**DOI:** 10.1038/s41418-025-01657-7

**Published:** 2026-01-08

**Authors:** Nana Chen, Mouxiang Fang, Leqi Zhong, Xiaolong Li, Yijia Zhou, Jianhua Zhan, Manli Wang, Zhaoyuan Fang, Hua Wang, Shijie Tang, Fang Liu, Bing Deng, Ning Chen, Jie Lei, Yuchen Zhang, Min Yan, Zhengzhi Zou, Yijun Gao, Chong Chen, Wenzhao Zhong, Srinivas Vinod Saladi, Hongbin Ji, Quentin Liu, Zifeng Wang, Bin He

**Affiliations:** 1 State Key Laboratory of Oncology in South China, Guangdong Provincial Clinical Research Center for Cancer, Psychobehavioral Cancer Research Center, Sun Yat-sen University Cancer Center, Guangzhou, China; 2 Guangdong Lung Cancer Institute, Guangdong Provincial Key Laboratory of Translational Medicine in Lung Cancer, Guangdong Provincial People’s Hospital, Guangdong Academy of Medical Sciences, Southern Medical University, Guangzhou, China; 3 MOE Key Laboratory of Laser Life Science & Guangdong Provincial Key Laboratory of Laser Life Science, College of Biophotonics, South China Normal University, Guangzhou, China; 4 Institute of Cancer Stem Cell, Dalian Medical University, Dalian, China; 5 State Key Laboratory of Cell Biology, Shanghai Institute of Biochemistry and Cell Biology, Center for Excellence in Molecular Cell Science, Chinese Academy of Sciences, Shanghai, China; 6 State Key Laboratory of Biotherapy and Cancer Center, West China Hospital, Sichuan University, Chengdu, China; 7 Department of Colorectal Surgery and Oncology, the Second Affiliated Hospital, and Center for Biomedical Systems and Informatics, Zhejiang University-University of Edinburgh Institute (ZJU-UoE Institute), Zhejiang University School of Medicine, Zhejiang University, Hangzhou, China; 8 Shanghai General Hospital, Shanghai Jiao Tong University School of Medicine, Shanghai, China; 9 Department of Cell and Cancer Biology, University of Toledo, College of Medicine and Life Sciences, Toledo, OH USA; 10 School of Medicine, Westlake University, Hangzhou, China

**Keywords:** Non-small-cell lung cancer, Epigenetics, Predictive markers

## Abstract

Lineage plasticity in non-small cell lung cancer (NSCLC) drives resistance to tyrosine kinase inhibitor (TKI) therapies, yet the epigenetic drivers of this phenotypic transition remain poorly defined. Here, we identify loss of the histone methyltransferase KMT2D as a critical event that disrupts adenocarcinoma lineage fidelity and promotes squamous transition. KMT2D expression is markedly reduced in TKI-resistant NSCLC with squamous-like features, and its mutation correlates with elevated squamous lineage markers and poorer clinical outcomes. Mechanistically, KMT2D loss triggers global transcriptional and epigenomic reprogramming, upregulating squamous master regulators such as ΔNp63 and SOX2. CRISPR-based screening reveals that KMT2D-deficient tumors are preferentially dependent on AURKA to maintain squamous identity and cell proliferation. Notably, loss of KMT2D enhances AURKA stability and activity by disrupting its interaction with the E3 ligase FBXW7, resulting in reduced ubiquitination and prolonged AURKA signaling. Pharmacologic inhibition of AURKA abrogates squamous features and suppresses tumor growth in patient-derived organoids, xenografts, and orthotopic models, with KMT2D-deficient tumors exhibiting heightened sensitivity. These findings uncover that KMT2D alteration drives chromatin reprogramming that facilitates adeno-to-squamous transition and identifies AURKA as a lineage-specific vulnerability, providing a precision strategy to overcome TKI resistance.

**Statement of significance**

Our study identifies KMT2D loss as a key event of lineage switch that promotes adeno-to-squamous transition and TKI resistance in NSCLC. This epigenetic shift renders tumors dependent on AURKA, revealing a novel therapeutic target to counteract drug resistance and improve treatment outcomes.

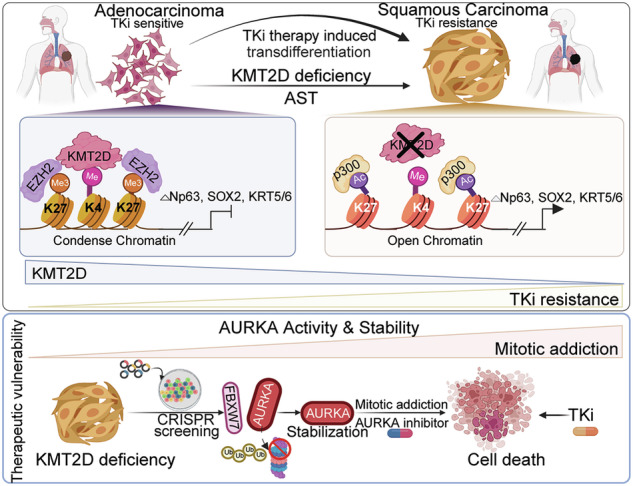

## Introduction

Lung cancer, particularly non-small cell lung cancer (NSCLC), remains a leading cause of cancer-related mortality globally [1], with lung adenocarcinoma (ADC) and lung squamous cell carcinoma (SCC) as its predominant subtypes [2]. The advent of targeted therapies, most notably epidermal growth factor receptor (EGFR) tyrosine kinase inhibitors (TKIs) such as osimertinib, has substantially improved clinical outcomes in EGFR-mutant NSCLC [3]. However, despite high initial response rates, virtually all patients ultimately relapse, highlighting therapeutic resistance as a critical barrier to durable disease control.

One clinically significant mechanism of resistance is lineage plasticity, whereby tumor cells escape targeted therapy not through secondary genetic mutations alone, but by adopting an alternative cell fate [4, 5]. In NSCLC, lineage plasticity is exemplified by a histological transformation in which lung adenocarcinoma evolves into a squamous cell carcinoma transition following tyrosine kinase inhibitor (TKI) treatment [6, 7]. This adeno-to-squamous transition (AST) is observed in approximately 2-10% of lung cancer patients and is associated with aggressive progression and lack of effective salvage options [7–9]. Importantly, patients with squamous transition often exhibit resistance to subsequent lines of therapy, underscoring that AST is not merely a phenotypic alteration but a clinically relevant driver of therapeutic failure [10, 11].

Mechanistically, AST represents a profound cellular reprogramming process in which LUAD cells progressively lose adenocarcinoma-defining features (e.g., NKX2-1, FOXA1, FOXA2) [12, 13] and acquire squamous lineage markers (e.g., ΔNp63, SOX2, KRT5/6A) [14–16]. Previous studies have identified genetic drivers such as loss of tumor suppressor LKB1, which destabilize adenocarcinoma identity and facilitate this switch [17]. Moreover, integrated analyses of genetic landscapes from pre- and post-transition clinical lung cancer specimens highlight the pivotal role of transcriptional reprogramming in lineage plasticity, particularly involving the AKT, MYC, PRC2, and RAPGEF3 signaling pathways [6, 18, 19]. These findings highlight that lineage plasticity is not solely a consequence of fixed genetic alterations but also heavily shaped by transcriptional and chromatin dynamics.

Indeed, tumor cell identity is fundamentally governed by the epigenetic landscape, which integrates oncogenic signaling with transcriptional networks [20]. Epigenetic regulators modulate enhancer-promoter interactions, chromatin accessibility, and histone modifications to stabilize lineage programs or enable lineage switching under selective pressure [21–23]. Foundational work on super-enhancers and cell-type-defining cis-regulatory circuits shows that master transcription factors cooperate with co-activators and chromatin remodelers to maintain identity genes in a high-output state [24–26]. In the context of TKI therapy, epigenetic remodeling may confer the plasticity that enables LUAD cells to adopt a squamous phenotype and survive therapeutic pressure. Yet the specific epigenetic regulators that orchestrate AST and drive TKI resistance remain incompletely defined. This knowledge gap hinders our understanding of lung cancer biology and limits the development of targeted therapies to overcome resistance. Clarifying the epigenetic governance of AST, spanning enhancer reprogramming, chromatin remodeling, and histone modifying, will be essential to map lineage dynamics in NSCLC and expose targetable dependencies underlying TKI resistance. Such insights will enable the design of rational strategies to block plasticity-driven resistance, ultimately improving outcomes for patients with EGFR-mutant lung cancer.

In this study, we identify KMT2D as a critical epigenetic regulator that preserves lung adenocarcinoma identity and maintains TKI sensitivity in EGFR-mutant NSCLC. Loss of KMT2D disrupts this lineage fidelity, driving squamous transition that underpins TKI resistance. Leveraging integrative transcriptomic and epigenomic profiling in combination with multiple experimental platforms, including lung cancer cell-derived xenografts, orthotopic grafts, patient-derived cell (PDC) models, and clinical specimens, we demonstrate that KMT2D loss rewires the epigenetic landscape to activate squamous transition programs while concurrently stabilizing the mitotic kinase AURKA by impairing FBXW7-mediated ubiquitination and degradation. This AURKA-dependent survival pathway creates a therapeutic vulnerability, as pharmacologic AURKA inhibition selectively suppresses KMT2D-deficient tumors, offering a precision strategy to overcome AST-associated TKIs resistance.

## Results

### Low KMT2D expression correlates with TKIs resistance and squamous transition in NSCLC

**Fig. 1:**
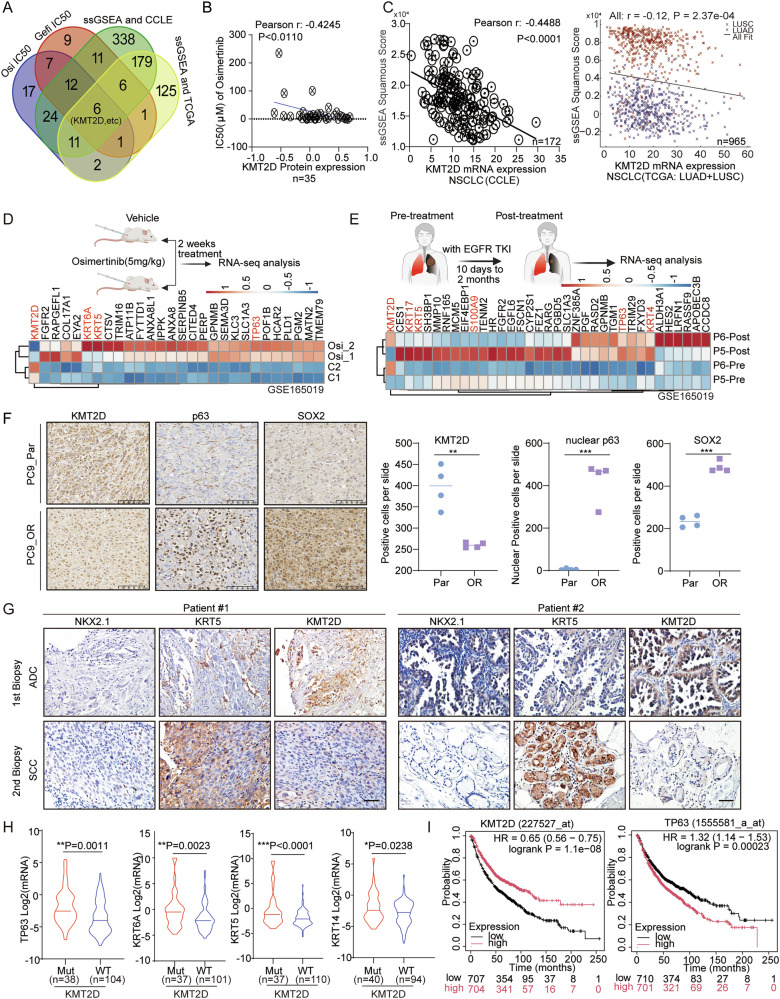
KMT2D low expression is associated with tyrosine kinase inhibitor (TKI) resistance and squamous phenotype transition. **A** Venn diagram showing the intersection of four datasets: genes negatively correlated with osimertinib IC_50_ (blue, n = 79, *P* < 0.05), gefitinib IC_50_ (red, n = 69, *P* < 0.05), genes negatively correlated with ssGSEA-derived squamous signature scores in CCLE lung cancer cell lines (green, n = 586, *P* < 0.05), and negatively correlated with ssGSEA-derived squamous signature scores in TCGA LUAD and LUSC tumors (yellow, n = 331, *P* < 0.05). A total of six genes, including KMT2D, were commonly identified across all datasets. **B** Pearson correlation between KMT2D protein expression and osimertinib IC_50_ in 35 NSCLC cell lines (r = −0.4245, *P* < 0.0110). **C** Left: Correlation analysis between KMT2D mRNA expression and the ssGSEA squamous signature score in CCLE NSCLC cell lines (n = 172, r = −0.4488, *P* < 0.0001). Right: Correlation analysis between KMT2D mRNA expression and the ssGSEA squamous signature score in TCGA lung cancer samples (n = 965; LUAD and LUSC; r = −0.1181, *P* < 0.0002). **D** Heatmap showing RNA-seq analysis of gene expression changes in xenograft tumors from mice treated with vehicle control or osimertinib (5 mg/kg) for 2 weeks (datasets from GSE165019). **E** Heatmap illustrating RNA-seq-based analysis of gene expression profiles from patient tumor samples collected pre- and post-treatment with EGFR tyrosine kinase inhibitors (TKIs). Each row represents a patient sample pair, and genes highlighted in red denote key squamous markers significantly altered following EGFR-TKI treatment (datasets from GSE165019). **F** Representative immunohistochemistry (IHC) images of xenograft tumors derived from PC9 parental (Par.) and osimertinib-resistant (OR) cells stained for KMT2D, p63, and SOX2. Scale bar, 100 µm. Right: Quantification of marker expression (n = 4). Two-tailed unpaired t-tests, **P < 0.01, **P < 0.001. **G** Representative immunostaining of indicated proteins in two paired human EGFR-mutant lung cancer specimens experiencing squamous transition after EGFR TKI failure (pre-1st biopsy vs. post-transition 2nd biopsy). Scale bar, 50 µm. **H** Violin plots showing the expression of squamous-associated genes in NSCLC cell lines from the CCLE, stratified by KMT2D mutation status (wild-type versus mutant). Two tailed t-test for Mut vs WT per gene (*P <  0.05, **P  <  0.01, ***P  <  0.001). **I** Kaplan–Meier survival curves were generated to assess the relationship between KMT2D and TP63 expression levels and the overall survival probability in lung cancer patients.

Emerging evidence suggests that squamous transition enables tumor cells to evade TKI therapies through lineage plasticity [10, 13, 27]. To systematically uncover epigenetic regulators that mediate lineage plasticity and confer resistance to EGFR-targeted therapies, we performed an integrative analysis combining pharmacogenomic profiling, gene expression datasets, and lineage-specific transcriptional signatures. We first correlated IC_50_ [28] values for first-generation (gefitinib) and third-generation (osimertinib) EGFR tyrosine kinase inhibitors (TKIs) with protein abundance data for ~1087 epigenetic regulators [29–31] across a panel of lung cancer cell lines. This analysis identified 69 and 79 epigenetic factors whose protein expression levels were significantly inversely correlated with TKIs sensitivity, respectively (Supplementary Table S1). To further prioritize candidate genes with squamous lineage reprogramming, we applied single-sample gene set enrichment analysis (ssGSEA) using two curated squamous differentiation signatures (10-gene and 35-gene sets) [10, 12, 13, 32] to score lung cancer cell lines from the CCLE [33] and primary tumor samples from TCGA [34] lung adenocarcinoma (LUAD, n = 494) and lung squamous cell carcinoma (LUSC, n = 471) cohorts. Correlation analysis between ssGSEA-derived squamous scores and the expression of 1,087 epigenetic regulators revealed that 349 genes (10-gene signature) and 586 genes (35-gene signature) were significantly negatively associated with squamous identity in NSCLC cells (Supplementary Table S1). In parallel, transcriptomic profiling in TCGA lung tumors identified 331 genes that inversely correlated with squamous scores (Supplementary Table S1). By intersecting four datasets, genes associated with resistance to first- and third-generation TKIs, negative regulators of the squamous signature in cell lines, and genes inversely correlated with the squamous program in patient lung tumors, we identified six candidate epigenetic regulators (Fig. 1A and Supplementary Table S1). Among these, KMT2D emerged as a prime candidate for further investigation due to its established role as a histone methyltransferase, catalyzing H3K4 monomethylation and contributing to enhancer activation and cell fate transitions [35, 36]. Consistent with this function, KMT2D protein abundance was inversely correlated with sensitivity to osimertinib and gefitinib across lung cancer cell lines (Figs. 1B, S1A and Supplementary Table S2). Furthermore, transcriptomic analyses revealed a significant inverse correlation between *KMT2D* expression and squamous lineage scores, as determined by single-sample gene set enrichment analysis (ssGSEA), in both CCLE lung cancer cell lines and the TCGA lung cancer cohort (Figs. 1C, S1B and Supplementary Table S2), suggesting that KMT2D may play a critical role in restraining squamous transition. Collectively, these multi-layered findings implicate KMT2D as a central epigenetic regulator linking lineage plasticity with therapeutic resistance in lung cancer.

To further assess the clinical relevance of our findings, we analyzed gene expression profiles using publicly available RNA-seq data from xenograft tumors treated with osimertinib (GSE165019) [37]. After 2 weeks of treatment, these tumors exhibited marked downregulation of *KMT2D*, along with upregulation of key squamous lineage markers, including *KRT6A*, *KRT5*, and *TP63* (Fig. 1D), supporting a potential link between KMT2D suppression and squamous transition. To determine whether KMT2D suppression is a conserved feature of TKI resistance, we analyzed matched pre- and post-treatment biopsies (duration 10 days-2 months) from EGFR-TKI-treated NSCLC patients [37]. Post-treatment tumors exhibited pronounced *KMT2D* downregulation alongside induction of squamous lineage markers (*KRT17*, *KRT5*, *TP63*) and basal-like factors (*S100A9*, *KRT4*) (Fig. 1E). This parallel evolution of resistance and phenotype switching mirrors our in vitro findings, positioning KMT2D loss as an important mediator linking TKI resistance to lineage reprogramming. This phenomenon may represent a previously unrecognized mechanism underlying therapeutic resistance.

To mechanistically validate this association, we established osimertinib-resistant NSCLC cell lines, PC9 osimertinib-resistant (PC9_OR) and H1975 osimertinib-resistant (H1975_OR) cells. These resistant cell lines exhibited a higher IC_50_ for osimertinib and almonertinib compared to their parental counterparts (Fig. S1C–F). RNA-seq analysis of osimertinib-resistant NSCLC cell lines revealed a transcriptional shift indicative of lineage plasticity. In PC9_OR cells, there was marked downregulation of adenocarcinoma-specific genes, including *NKX2-1*, *SFTPB*, *SFTPD*, and *MUC15* (Fig. S1G). Similarly, H1975_OR cells showed reduced expression of *NKX2-1*, *FOXA2*, *SFTA1P*, and *KRT7* (Fig. S1G). In parallel, both resistant models exhibited upregulation of squamous lineage markers such as *TP63*, *SOX2*, *KRT16*, *S100A8*, *COL7A1*, and *KRT17* (Fig. S1G). This gene expression pattern closely aligns with previously reported signatures of squamous differentiation in NSCLC [12], suggesting that resistance to EGFR inhibition may be coupled with a shift toward a squamous-like cell state. Additionally, western blot analysis confirmed a reduction in KMT2D expression in osimertinib-resistant cells, accompanied by elevated levels of p63 and SOX2 (Fig. S1H; Original data file 1). Our immunohistochemical (IHC) analysis of xenograft tumor sections derived from PC9 parental and osimertinib-resistant cells revealed a significant reduction in KMT2D expression in resistant tumors, accompanied by upregulation of p63 and SOX2 (Fig. 1F). To corroborate the association between KMT2D loss and lineage plasticity, we analyzed paired biopsy samples from patient’s pre-treatment and post-TKI resistance. IHC staining showed a marked decrease in KMT2D expression in post-TKI resistant tumors, coinciding with increased expression of the squamous marker KRT5 and decreased expression of the adenocarcinoma marker NKX2-1 (Fig. 1G), further supporting the role of KMT2D loss in adeno-to-squamous carcinoma transition. Consistent with these findings, IHC analysis of patient-derived squamous tumors revealed low KMT2D expression, accompanied by high levels of p63 and KRT5/6, which strengthens the link between KMT2D loss and squamous transition (Fig. S1I). Furthermore, patient-derived cells harboring KMT2D mutations exhibited robust upregulation of squamous carcinoma-associated genes, such as *TP63*, *KRT6A*, *KRT5*, and *KRT14* (Fig. 1H). Survival analysis in lung cancer patients revealed that low KMT2D expression is strongly correlated with poor overall survival (Fig. 1I), positioning KMT2D as a critical prognostic biomarker. In addition, high expression of squamous markers such as *TP63*, *SOX2*, *KRT5, and KRT6A* also predicts unfavorable outcomes in lung cancer patients (Figs. 1I and S1J). Collectively, these findings establish KMT2D loss as a key molecular event contributing to lineage plasticity and TKI resistance in NSCLC.

### KMT2D deficiency drives adeno-to-squamous transition through chromatin rewiring

**Fig. 2:**
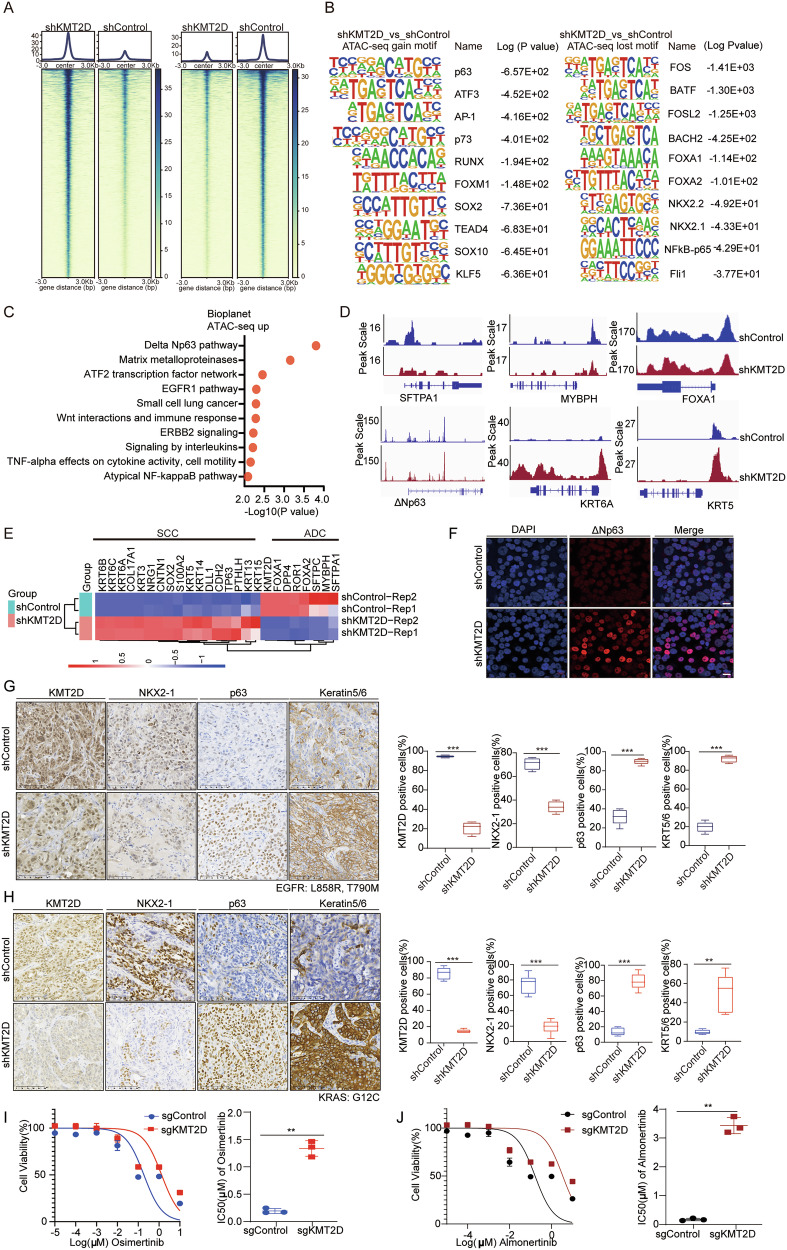
KMT2D deficiency drives lung adenocarcinoma to a squamous transition through epigenetic chromatin rewiring. **A** ATAC-seq analysis of differentially accessible chromatin in WT (shControl) and KMT2D (shKMT2D) deficient cells. Heatmap showing ATAC-seq signal intensity in a 3 kb region centered on TSS. **B** Motif enrichment analysis of ATAC-seq peaks in KMT2D knockdown versus control cells. Log P-value: Statistical significance of motif enrichment (log-transformed). **C** BioPlanet integrates pathway analysis of genes corresponding to up-regulated differential peaks identified by ATAC-seq in KMT2D knockdown versus control groups. **D** Integrative Genomics Viewer (IGV) tracks of ATAC-seq showing the normalized peak scale (vertical axis) of adenocarcinoma and squamous lineage related genes derived from Control and KMT2D knockdown group. **E** Heatmap of ADC- and SCC-related genes from RNA-seq analysis in shControl and shKMT2D cells. Expression values represented as Z score of Log2-transformed TPM. **F** Representative immunofluorescence staining of the ΔNp63 in shControl and KMT2D knockdown cells. Blue: DAPI (nuclei); Red: ΔNp63. Scale bar, 20 µm. **G**, **H** Representative IHC for KMT2D, NKX2-1, p63, and KRT5/6 in xenograft tumors with EGFR ^L858R/T790M^ or KRAS^G12C^ mutations (n = 5 tumors/group; 5–10 random HPFs per tumor averaged). Scale bar, 100 µm. Quantification shows the percentage of positive cells, data are mean ± SD with individual tumors overlaid. Group differences (shControl vs shKMT2D) were tested by unpaired two-tailed t-test, **P < 0.01, **P < 0.001. **I**, **J** Dose-response curves and IC₅₀ analysis of osimertinib or almonertinib treatment in H1975 cells transduced with either sgControl or sgKMT2D. Data are presented as mean ± SD from three independent experiments. Statistical significance was determined using unpaired two-tailed *t*-test, **P < 0.01.

Given that KMT2D functions as a histone H3K4 monomethyltransferase [35], we hypothesized that its loss would remodel chromatin landscapes to suppress adenocarcinoma identity and license squamous differentiation. ATAC-seq profiling of KMT2D-deficient cells revealed global chromatin reorganization, with distinct clustering of accessible regions (Fig. 2A). Mechanistically, motif enrichment analysis uncovered opposing transcription factor (TF) networks, regions of gained accessibility were dominated by squamous drivers (p63, SOX2) [38], while lost regions harbored adeno-specific regulators (FOXA1/2, NKX2-1) [39] (Fig. 2B). Strikingly, p63, the master squamous lineage TF, with its ΔNp63 isoform serving as the mediator of squamous identity [32], exhibited the strongest motif enrichment, positioning it as an important effector of KMT2D dependent plasticity. Functionally, the pathway enrichment analysis showed that the chromatin regions of increased accessibility were associated with ΔNp63 pathway, EGFR1 pathway, ERBB2 and atypical NF-κB signaling (Fig. 2C). These findings suggest that KMT2D knockdown alters chromatin accessibility by modulating the binding of key lineage-specific transcription factors, thereby reshaping the chromatin landscape to promote lung cancer plasticity. To directly connect chromatin remodeling to lineage switching, we assessed the impact of KMT2D knockdown on chromatin accessibility at gene loci associated with lung adenocarcinoma and squamous carcinoma lineage-specific markers. Our results showed that adenocarcinoma-related genes such as *SFTPA1*, *MYBPH* [40], and *FOXA1* exhibited reduced chromatin accessibility in KMT2D-deficient cells. In contrast, squamous-associated genes, including *ΔNp63*, *KRT6A*, *KRT5*, *KRT14*, *S100A2*, *PTHLH*, and *ITGB4* [32] exhibited increased accessibility (Figs. 2D and S2A). These changes in chromatin dynamics indicate that KMT2D loss reprograms the chromatin landscape to favor the activation of squamous lineage programs while repressing adenocarcinoma-associated genes.

To define the transcriptional consequences of KMT2D-dependent chromatin remodeling, we performed RNA-seq on KMT2D-depleted cells. Strikingly, KMT2D loss induced a bidirectional transcriptional shift: suppression of adenocarcinoma (ADC) identity genes (*FOXA1*, *FOXA2*, *ROR1*, *SFTPC*, *SFTPA1*, and *MYBPH*) [39, 41] coupled with activation of squamous cell carcinoma (SCC) markers (*KRT6*, *SOX2*, *KRT5*, *KRT14*, *TP63, S100A2*, and *PTHLH*) [10, 12, 32] (Figs. 2E and S2B). This reciprocal regulation aligns with our ATAC-seq data, indicating that KMT2D deficiency disrupts ADC identity and promotes a shift toward SCC characteristics. To further validate protein-level upregulation of squamous markers, immunofluorescence with antibodies to ΔNp63 (p40), p63, and KRT5/6 showed markedly increased signal intensities in KMT2D-deficient cells, consistent with the transcriptomic data (Figs. 2F and S2C, D). To validate these findings in vivo, we established xenograft models by subcutaneously injecting NSCLC cells harboring EGFR (L858R, T790M) or KRAS (G12C) mutations that were transduced with either control shRNA (shControl) or KMT2D-targeting shRNA (shKMT2D). IHC analysis confirmed efficient KMT2D knockdown, as indicated by a marked reduction in KMT2D staining in shKMT2D tumors (Fig. 2G, H). Notably, KMT2D-deficient tumors exhibited significantly increased expression of squamous lineage markers p63 and KRT5/6, accompanied by a pronounced reduction in the adenocarcinoma marker NKX2-1/TTF-1, indicative of a phenotypic shift toward a squamous-like state (Fig. 2G, H). Nuclear ΔNp63 induction was independently confirmed by p40 IHC and an additional ΔNp63 antibody, with concordant results (Fig. S2E, F). Furthermore, compared with shControl, shKMT2D xenografts exhibited a higher Ki-67 proliferative index, indicating increased proliferation concurrent with lineage reprogramming (Fig. S2G). To assess whether this lineage transition contributes to resistance to EGFR-targeted therapies, we performed dose-response analyses using third-generation TKIs. Knockout of KMT2D significantly reduced sensitivity to both osimertinib and almonertinib, further supporting its role in mediating AST associated drug resistance (Figs. 2I, J and S2H; Original data file 1). Collectively, our findings establish KMT2D as a critical epigenetic regulator of lineage fidelity in NSCLC. In adenocarcinoma cells, KMT2D promotes open chromatin at alveolar-specific loci such as FOXA1 and SFTPA1, while maintaining repressive chromatin at squamous-associated loci like ΔNp63 and KRT5/6. Loss of KMT2D leads to enhancer reprogramming, resulting in chromatin condensation at adenocarcinoma-specific regulatory elements and increased accessibility at squamous lineage enhancers (Fig. S2I). This shift in chromatin state facilitates phenotypic reprogramming toward a squamous-like identity and promotes resistance to EGFR-targeted therapies. These results provide mechanistic insight into how KMT2D deficiency drives lineage plasticity and therapeutic resistance in lung cancer.

### Loss of KMT2D interrupts the epigenetic crosstalk required to maintain lineage fidelity

**Fig. 3:**
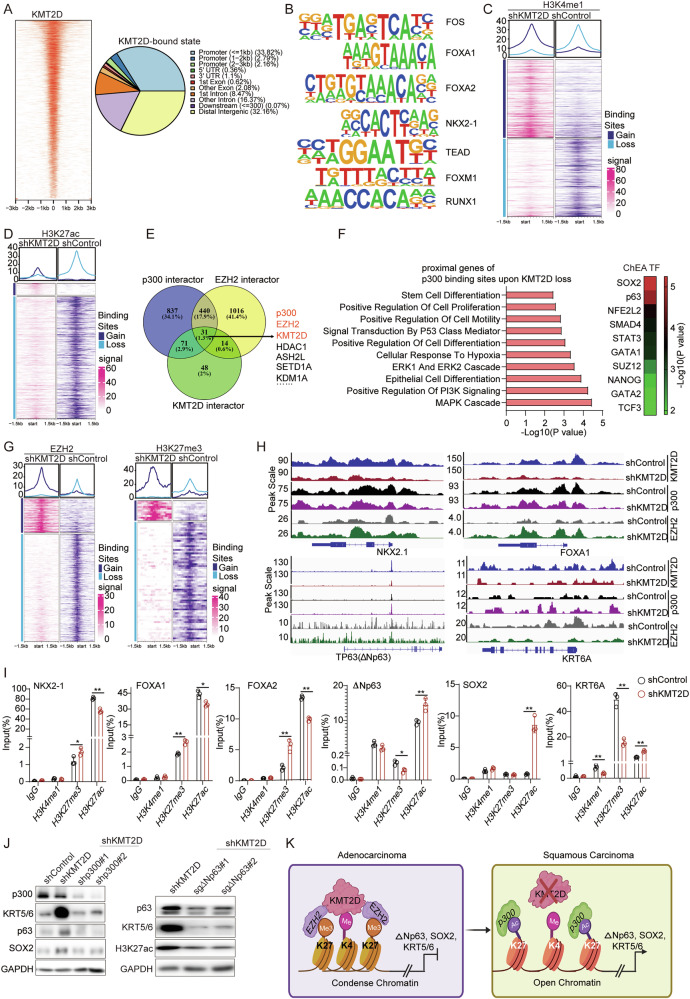
Loss of KMT2D interrupts the epigenetic crosstalk required to maintain lineage fidelity. **A** Genome-wide KMT2D occupancy. Left: Heatmap of normalized KMT2D signal centered on peak summits (±3 kb), peaks ranked by intensity. Right: Genomic annotation of KMT2D peaks. **B** De novo motif analysis at KMT2D, H3K4me1, and H3K27ac binding sites identified significant enrichment for motifs of AP-1 (FOS), FOXA1, FOXA2, NKX2-1, TEAD, FOXM1, and RUNX1. **C**, **D** Heatmaps and average profiles of H3K4me1 and H3K27ac signal in shKMT2D versus shControl cells centered on the analyzed genomic regions (±1.5 kb). **E** Venn diagram showing the overlap among p300, EZH2, and KMT2D interacting proteins from BioGRID. Numbers indicate interactor counts in each category; percentages are relative to the total interactome set. **F** Bar plot (left) showing enriched Gene Ontology (GO) terms among genes proximal to p300 peaks after KMT2D depletion. Heatmap (right) displays ChEA transcription-factor enrichment, colors indicate −log10(P). **G** Heatmaps and average profiles of EZH2 and H3K27me3 signal in shKMT2D and shControl cells centered on the analyzed regions (±1.5 kb). **H** IGV showing normalized CUT&Tag signal for KMT2D, p300, and EZH2 across promoter and enhancer regions of adenocarcinoma-associated genes (NKX2-1, FOXA1) and squamous-associated genes (ΔNp63, KRT6A). **I** ChIP-PCR analysis of enrichment of H3K4me1, H3K27me3, and H3K27ac at the NKX2-1, FOXA1, FOXA2, ΔNp63, SOX2, and KRT6A promoter or enhancer, expressed as % input. Data are presented as mean ± SD (n = 3). Group differences were assessed by multiple unpaired two-tailed t-tests (one comparison per mark/locus). IgG serves as the negative control. *P < 0.05; **P < 0.01. **J** Western blot analysis of squamous lineage markers in KMT2D-deficient cells following p300 knockdown or ΔNp63 depletion. **K** Model of KMT2D-dependent regulation of lineage-specific chromatin states in NSCLC via EZH2 or p300 (Created with BioRender.com).

Since KMT2D plays a critical and multifaceted role in regulating gene expression by modifying chromatin structure at both promoters and enhancers to affect cell fate [36]. To further map its epigenomic role in lung adenocarcinoma, we performed CUT&Tag profiling for KMT2D and the enhancer marks H3K4me1 and H3K27ac. Approximately ~39% of KMT2D peaks were promoter-proximal and ~32% mapped to distal intergenic regions (Fig. 3A). Consistent with its enhancer priming role, H3K4me1 mainly occupied distal sites (~40%), whereas H3K27ac spanned both promoters (~29%) and distal intergenic region (~33%) (Fig. S3A, B). Motif enrichment analysis of chromatin regions co-occupied by KMT2D, H3K4me1, and H3K27ac revealed significant enrichment of transcription factor motifs associated with lung adenocarcinoma epithelial identity, including AP-1 (FOS), FOXA1, FOXA2, and NKX2-1, as well as oncogenesis-associated factors such as TEAD, FOXM1, and RUNX1 (Figs. 3B and S3C) [39, 42–45]. The presence of these motifs within KMT2D-bound regulatory elements highlights KMT2D’s important role in preserving enhancer landscapes that maintain lineage fidelity and support oncogenic transcriptional programs in lung adenocarcinoma. To assess the broader impact of KMT2D loss, we performed differential peak analysis of H3K4me1 and H3K27ac following KMT2D knockdown. This analysis revealed significant gains and losses of these histone marks across numerous genomic regions (Figs. 3C, D and S3D), suggesting that KMT2D depletion disrupts normal enhancer and promoter function, implying locus-specific rather than global changes in H3K4me1 and H3K27ac. Given KMT2D’s role in recruiting H3K27 acetyltransferases CBP and p300 [46, 47], we explored its interactions via the BioGRID database and confirmed the association between KMT2D and p300 by co-immunoprecipitation (Figs. 3E,S3E and Supplementary Table S3; Original datafile 1). Based on these findings, we hypothesized that loss of KMT2D would compromise promoter and enhancer integrity, leading to extensive reprogramming of the transcriptional landscape. To test this, we profiled p300 binding using CUT&Tag. Heatmaps of p300 signal centered on peaks showed a redistribution of p300 upon KMT2D depletion, with site-specific gains and losses indicative of broad epigenetic reprogramming (Fig. S3F). Gene Ontology (GO) analysis of regions with altered p300 occupancy revealed enrichment for pathways linked to cell differentiation, and proliferation, consistent with the observed phenotypic shifts (Fig. 3F). Notably, p300-bound regions following KMT2D loss were most enriched for SOX2 and p63 motifs, implicating these factors in activating a squamous-like transcriptional program (Fig. 3F) [38, 48, 49]. Previous studies have reported that KMT2D interacts with the H3K27me3 methyltransferase EZH2, suggesting a dual role for KMT2D in coordinating chromatin activation and repression [50–52]. Consistent with these findings, we also observed an interaction between KMT2D and EZH2 in our study (Fig. S3E and S3). CUT&Tag analysis of EZH2 and H3K27me3 binding regions revealed a more extensive loss of repressive H3K27me3 marks compared to regions showing gains after KMT2D depletion, reflecting the dynamic and unbalanced epigenomic remodeling triggered by KMT2D loss (Fig. 3G). Genome browser (IGV) profiles revealed distinct epigenetic changes at representative gene loci. In adenocarcinoma regulators such as *NKX2-1* and *FOXA1*, KMT2D loss led to reduced p300 binding and increased EZH2 occupancy, consistent with transcriptional repression (Fig. 3H). In contrast, squamous-associated genes, such as *ΔNp63* and *KRT6A*, exhibited increased p300 binding and reduced EZH2 occupancy, supporting their transcriptional activation in the absence of KMT2D (Fig. 3H). In parallel, quantitative analysis revealed that KMT2D loss led to a marked reduction in H3K27ac levels at the promoters or enhancers of adenocarcinoma lineage markers (NKX2-1, FOXA1, and FOXA2), accompanied by an increase in H3K27me3 deposition, indicative of a transcriptionally repressive chromatin state (Fig. 3I). In contrast, increased H3K27ac at enhancer or promoter regions of squamous lineage genes (ΔNp63, SOX2, and KRT6A), together with reduced H3K27me3 at the ΔNp63 and KRT6A loci, indicates a shift toward an active chromatin state, supporting enhanced transcriptional activation of the squamous program (Fig. 3I). Moreover, p300 knockdown led to reduced expression of p63, SOX2, and KRT5/6 (Fig. 3J; Original data file 1), indicating its role in maintaining the squamous transcriptional program. To establish the mechanistic link between p300 and the key squamous factor ΔNp63, we performed co-immunoprecipitation and confirmed a direct physical interaction between them (Fig. S3G; Original data file 1). This interaction provides a molecular basis for the observed functional linkage, as ΔNp63 knockout similarly decreased KRT5/6 expression (Fig. 3J; Original data file 1). Together, the data indicate that ΔNp63 acts not only downstream of p300 but also in complex with p300 to drive squamous-lineage gene expression. Together, our findings reveal that KMT2D deficiency enables p300 driven activation of squamous-specific regulatory elements, including ΔNp63 and SOX2, thereby promoting squamous transition and resistance to TKI therapy in NSCLC (Fig. 3K).

### KMT2D deficiency generates a mitotic kinase vulnerability in NSCLC

**Fig. 4:**
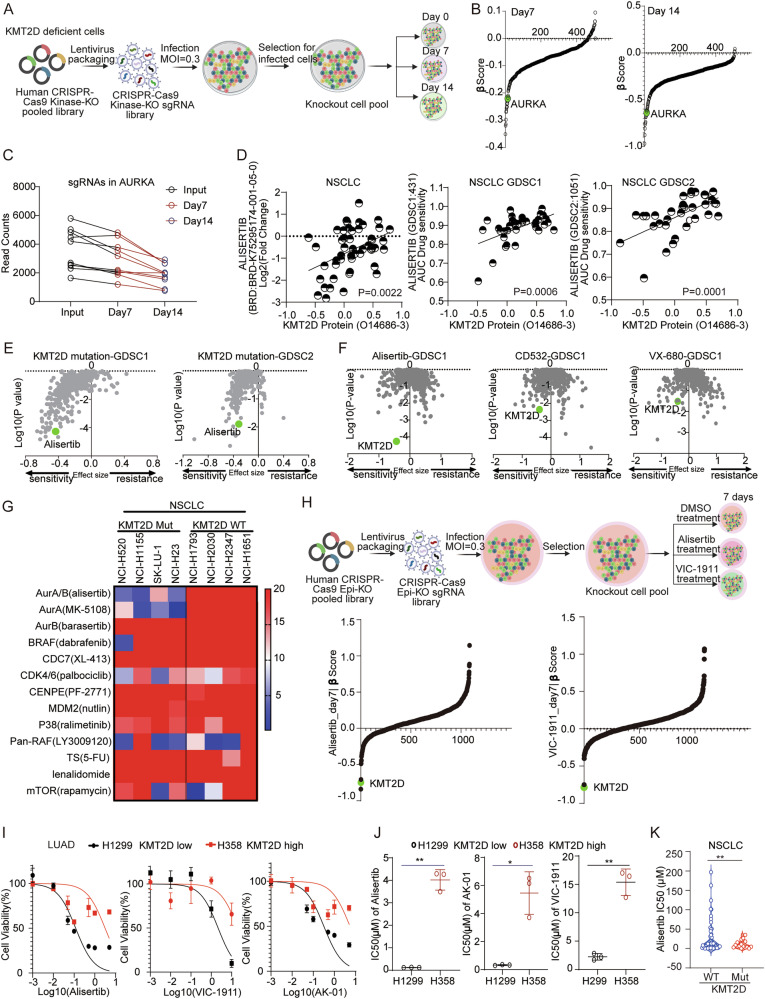
Kinome-wide CRISPR-Cas9 knockout screens revealed AURKA as a therapeutic target for KMT2D deficient lung cancer. **A** Experimental flow chart showing kinome-wide CRISPR-Cas9 knockout screening in KMT2D deficient cells (Created with BioRender.com). **B** β scores for gene essentiality were calculated following 7-day or 14-day screening periods. **C** The normalized counts of sgRNA targeting AURKA in day 0, day 7, and day 14 in KMT2D deficient lung cancer cells. **D** Scatter plots showing the correlation between KMT2D protein expression and alisertib sensitivity in non-small cell lung cancer (NSCLC). Data were analyzed based on different datasets from DepMap Portal. *X*-axis indicates the relative expression level of the protein. *Y*-axis indicates the log2 (fold change) and AUC (Area Under the Curve) value of the inhibitors. **E** Scatterplots indicate the effect size of the inhibitors from drug-screening analysis. Green indicates AURKA inhibitors, which are the most effective inhibitors in KMT2D-mutant cancer cells. Data from the GDSC1 and GDSC2 (Genomics of Drug Sensitivity in Cancer project phase 1 and phase 2). **F** Scatterplots show the effect size of AURKA inhibitors (Alisertib, CD532, and VX-680) across various pan-cancer cell lines with specific genomic mutations. Green dots represent the effect size in cell lines with KMT2D mutations. Data are sourced from the GDSC1 (Genomics of Drug Sensitivity in Cancer project, Phase 1). **G** Heatmap depicting the differential sensitivity of non-small cell lung cancer (NSCLC) to various drugs, stratified by KMT2D mutation status. Drug sensitivity is represented as a color gradient, where red indicates low sensitivity (higher IC_50_ or lower drug efficacy) and blue indicates higher sensitivity (lower IC_50_ or high drug efficacy). **H** Flowchart showing CRISPR KO-based dropout screening in DMSO and AURKA inhibitors (alisertib and VIC-1911) treated-cells (Created with BioRender.com). Rank plot showing the distribution of β scores of the genes were identified as essential in AURKA inhibitor treatment group. **I** Dose-response curves illustrating the effect of AURKA inhibitors (Alisertib, VIC1911 and AK-01) on cell viability in KMT2D-high and KMT2D-low expression lung cell lines. **J** Dose-response curves illustrating the effect of AURKA inhibitors (alisertib, VIC-1911, AK-01) on cell viability in KMT2D-high and KMT2D-low lung squamous cell lines. **K** Violin plots showing the distribution of IC_50_ values for alisertib in NSCLC with wild-type (WT) and mutant (Mut) KMT2D. Two-tailed unpaired t test. **P < 0.01. IC₅₀ data were obtained from GDSC2, and KMT2D status was assigned based on CCLE lung cancer cell line annotations.

To identify druggable kinase dependencies in KMT2D-deficient lung cancer, we performed CRISPR-Cas9 loss-of-function screens using a custom sgRNA library targeting 507 kinases across the human kinome [53]. The KMT2D-deficient cells were first transduced with lentiviral sgRNAs and then subjected to puromycin selection, ensuring only transduced cells remained. We determined baseline sgRNA representation at day 0 and collected samples at days 7 and 14 to identify sgRNAs that became depleted over time indicating the loss of genes essential for the viability of KMT2D-deficient cells (Fig. 4A). Gene set enrichment analysis (GSEA) of candidate kinases revealed significant enrichment in pathways involved in the mitotic cell cycle, G2/M phase transition, and cell cycle checkpoints (Fig. S4A). Differential β-score analysis allowed us to distinguish sgRNAs that were negatively or positively selected, indicating kinases whose loss either impairs or enhances cell viability, respectively. Among the top negatively selected kinases at both time points were PLK1, CDK9, CHEK1, and AURKA, all of which are currently targeted by inhibitors under clinical evaluation (Fig. 4B, C and Supplementary Table S4). To refine the selection of candidate targets, we integrated genomic and proteomic datasets with drug sensitivity profiles from the Genomics of Drug Sensitivity in Cancer (GDSC) database [28]. This integrative analysis demonstrated that KMT2D-mutant cells exhibited heightened sensitivity to AURKA inhibitors, such as alisertib and GSK1070916, compared to other kinase inhibitors (Fig. S4B). To assess the specificity of these hits as vulnerabilities upon KMT2D deficiency, we interrogated DepMap datasets. Notably, KMT2D protein levels were inversely correlated with sensitivity to AURKA inhibitors, indicating that cells with lower KMT2D expression are more responsive to AURKA-targeted therapies (Fig. 4D). This association was not observed for inhibitors targeting PLK1, CDK9, or CHEK1, suggesting a selective dependency on AURKA in the context of KMT2D loss (Fig. S4C). Moreover, KMT2D mutations were significantly enriched among genomic features predictive of sensitivity to multiple AURKA inhibitors, including alisertib, CD532, and VX-680 (Fig. 4E, F), consolidating KMT2D loss as a potential biomarker for stratifying NSCLC patients benefiting from AURKA inhibition. Pharmacogenomic screens across NSCLC and SCLC cell lines [54] reinforced this finding, demonstrating that KMT2D mutations correlated with heightened sensitivity to all tested AURKA inhibitors (Figs. 4G and S4D), highlighting a therapeutic window for KMT2D-mutant cancers.

To establish bidirectional evidence for KMT2D loss as a driver of AURKA inhibitor sensitivity, we performed a secondary CRISPR-Cas9 screen using a focused sgRNA library targeting epigenetic regulators (Supplementary Table S4). NSCLC cells were transduced at low multiplicity of infection (MOI = 0.3), selected with puromycin, and treated with alisertib, VIC-1911, or DMSO control for 7 days (Fig. 4H). Strikingly, β-score analysis confirmed that KMT2D knockout (sgKMT2D) exhibited the strongest synergistic lethality with AURKA inhibitors (Figs. 4H and S4E), recapitulating our primary screen results and DepMap-derived correlations and establishing KMT2D loss as a bona fide sensitizer. To experimentally validate these findings, we evaluated the responses of KMT2D-high (H358, H2170) and KMT2D-low (H1299, SK-MES-1) NSCLC cell lines to a panel of AURKA inhibitors (alisertib, VIC-1911, AK-01, CD532) and an AURKA-targeting degrader, dAurK383, which was developed in our laboratory [55]. Dose-response assays revealed that KMT2D-low cells were significantly more sensitive to AURKA inhibition, as reflected by markedly lower IC_50_ values (Figs. 4I, J and S4F–I; Original data file 1). This finding underscores the critical role of KMT2D in regulating cellular response to AURKA-targeted therapies. Taken together, these data identify AURKA as a key therapeutic vulnerability in KMT2D-deficient lung cancer, providing a rationale for the clinical exploration of AURKA inhibitors as a targeted treatment strategy for KMT2D-mutant tumors.

To evaluate the translational relevance of this dependency, we stratified lung cancer cell lines by KMT2D status and compared their sensitivity to the AURKA inhibitor alisertib, KMT2D-mutant lung cancer cell lines exhibited lower alisertib IC₅₀ values than KMT2D wild-type lines, with a significant difference in NSCLC (LUAD and LUSC) (Fig. 4K). Notably, KMT2D deficiency has also been associated with resistance to EGFR inhibitors such as osimertinib, suggesting that AURKA inhibition may represent a rational therapeutic strategy. Collectively, these findings establish AURKA as a genotype-specific vulnerability in KMT2D-deficient lung cancer, supporting its potential as a therapeutic target in this subset of patients.

### KMT2D loss sensitizes NSCLC to AURKA inhibition

**Fig. 5:**
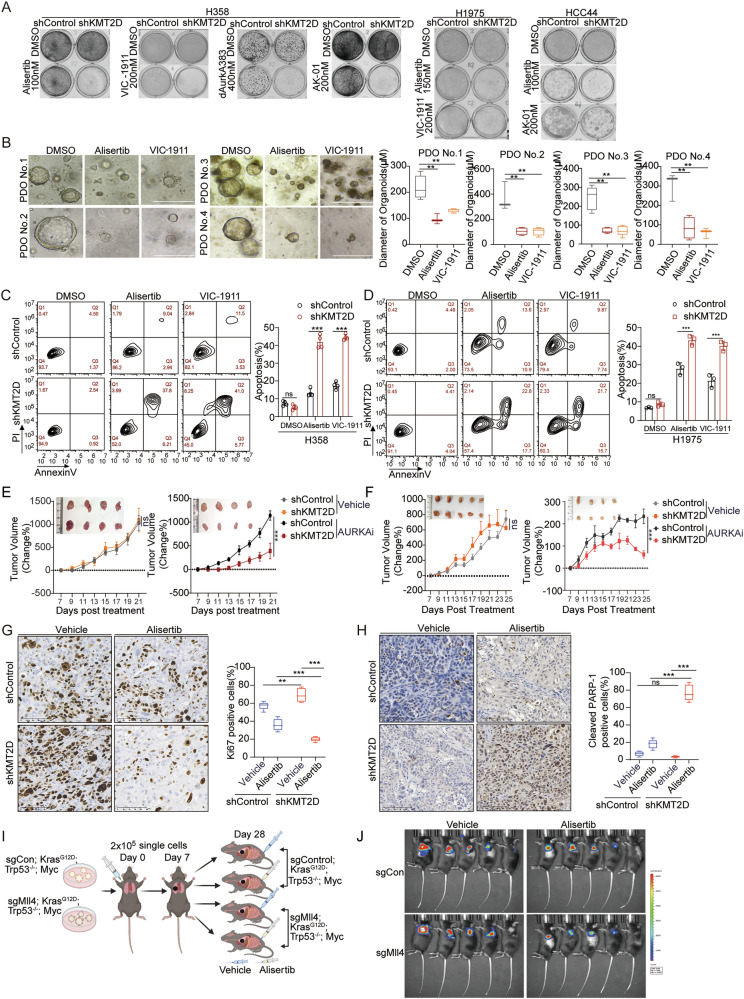
KMT2D loss confers vulnerability to AURKA inhibition in vitro and in vivo. **A** Representative colony-formation images for NSCLC cells with shControl or shKMT2D under treatment with the indicated AURKA inhibitors. **B** Right: Representative images of patient-derived organoids (PDOs) after AURKA inhibitor treatment (Alisertib, VIC-1911). Scale bars, 500 μm. Left: Diameter quantification of PDOs under the indicated conditions (mean ± SD, n = 4 or 5). One-way ANOVA with Dunnett’s multiple-comparisons test versus DMSO. **P < 0.01. **C**, **D** Apoptosis analysis of various NSCLC cells after AURKA inhibitor treatment. The cells in each group were stained with APC-conjugated Annexin V and PI and analyzed by flow cytometry. The numbers in box represents the cell proportion in each quadrant. The statical analysis were did in each group. Data shown as mean ± SD with individual points (n = 3 or 4). Two-way ANOVA. ***P < 0.001; ns not significant. **E**, **F** Normalized tumor volume for H1975 and H358 xenografts (shControl vs. shKMT2D) treated with vehicle or alisertib (30 mg/kg). Values are % change from treatment baseline; *x*-axis shows days on treatment. Data are mean ± SD (n = 4, 5 mice/group). Statistical comparisons were performed using two-way ANOVA. ***P < 0.001; ns not significant. **G** Left: Representative images show Ki67-positive nuclei (brown staining) in xenograft tumor sections derived from cells expressing either control shRNA (shControl) or shRNA targeting KMT2D (shKMT2D), treated with vehicle or the AURKA inhibitor alisertib. Scale bars: 100 µm. Right: The box plot (right) quantifies the percentage of Ki67-positive cells in each group (n = 4, 5; 5–10 random HPFs per tumor averaged). Two-way ANOVA test, **P < 0.01, ***P < 0.001. **H** Left: Representative images of cleaved-PARP1 staining in xenograft tumor sections derived from cells expressing either control shRNA (shControl) or shRNA targeting KMT2D (shKMT2D), treated with vehicle or the AURKA inhibitor alisertib. Scale bars: 100 µm. Right: The box plot (right) quantifies the percentage of cleaved-PARP1-positive cells in each group (mean ± SD, n = 4, 5 tumors per group; 5–10 random HPFs per tumor averaged). Two-way ANOVA test, **P < 0.01, ***P < 0.001; ns not significant. **I** Schematic overview of orthotopic mice model (Created with BioRender.com). **J** Representative IVIS Lumina images of *Kras*^*G12D*^; *Trp53*^*−/−*^; *Myc* mice with sgControl or sgMll4/Kmt2d tumors under vehicle or alisertib treatment (day 7–28). Color scale indicates radiance, identical exposure and scaling were used across groups.

Building on our observation that KMT2D-mutant lung cancer cells display heightened sensitivity to AURKA inhibitors, we next sought to functionally validate whether KMT2D loss directly modulates this drug response. To this end, cells were treated with a panel of AURKA inhibitors, including the selective inhibitors alisertib and VIC-1911, as well as the AURKA degrader dAurK383. In all conditions, KMT2D-deficient cells showed increased sensitivity, exhibiting reduced viability and significantly lower IC_50_ values compared to shControl cells (Fig. S5A–C; Original data file 1). Consistently, across NSCLC models (H358, H1975, HCC44), KMT2D knockdown markedly diminished clonogenic survival upon AURKA targeting (alisertib, VIC-1911, dAurK383, AK-01), confirming that KMT2D loss heightens vulnerability to AURKA-targeted therapy (Figs. 5A and S5A). Together, these data establish a causal relationship between KMT2D deficiency and increased AURKA inhibitor sensitivity in lung cancer cells. To test clinical relevance, we exposed patient-derived lung cancer organoids (PDOs) from four independent patients with the AURKA inhibitors alisertib and VIC-1911. Both inhibitors significantly reduced organoid size compared to DMSO controls, indicating impaired growth and supporting the efficacy of AURKA inhibition in patient-derived models (Fig. 5B). To assess whether the enhanced sensitivity conferred by KMT2D loss involves apoptosis, we performed Annexin V/PI staining in a panel of lung cancer cell lines with stable KMT2D knockdown. Treatment with alisertib or VIC-1911 significantly increased apoptotic cell populations in shKMT2D cells compared to controls (Figs. 5C, D and S5D, E), indicating that KMT2D loss sensitizes cancer cells to AURKA inhibition through apoptotic induction.

To further validate these findings in vivo, we established subcutaneous xenograft models using lung cancer cells with KMT2D knockdown. Mice were treated with either vehicle or the AURKA inhibitor alisertib, and tumor growth was monitored over time. While vehicle-treated shControl tumors showed progressive growth, alisertib treatment significantly suppressed tumor progression in KMT2D-deficient xenografts (Figs. 5E, F and S5F). These results demonstrate that KMT2D loss enhances the antitumor efficacy of AURKA inhibition in vivo, supporting the potential of AURKA-targeted therapy in KMT2D-deficient tumors. In xenograft tumors, vehicle-treated shKMT2D samples exhibited robust Ki67 staining, indicating high proliferative activity. Alisertib treatment substantially decreased Ki67-positive cells in shKMT2D tumors, demonstrating effective growth suppression in the context of KMT2D loss (Fig. 5G). Cleaved PARP-1 staining further showed increased apoptosis in alisertib-treated shKMT2D tumors compared to controls, highlighting that AURKA inhibition enhances apoptosis in the context of KMT2D deficiency (Fig. 5H). These findings collectively demonstrate that loss of KMT2D sensitizes tumors to alisertib by simultaneously reducing proliferation and inducing apoptosis. To evaluate the clinical potential of alisertib, we used an immunocompetent orthotopic mouse model by injecting *Kras*^*G12D*^*; Trp53*^*−/−*^*; Myc* tumor cells with or without Kmt2d (Mll4) knockout into the lungs of syngeneic mice (Fig. S5G; Original data file 1). Alisertib treatment, initiated on day 7 post-injection, significantly reduced tumor burden in sgMll4 mice, as measured by IVIS bioluminescence imaging (Fig. 5I, J). H&E staining confirmed reduced tumor size in alisertib-treated Kmt2d-deficient tumors (Fig. S5H). Consistently, Ki67 staining showed decreased proliferation, highlighting the efficacy of AURKA inhibition in Kmt2d-deficient lung tumors (Fig. S5H). Collectively, these in vivo data underscore the enhanced therapeutic efficacy of alisertib in Kmt2d-deficient tumors, supporting AURKA as a clinically actionable target in this genetically defined subset. These findings reinforce the concept that targeting AURKA can suppress tumor growth and phenotypic plasticity associated with KMT2D deficiency in lung cancer.

### KMT2D loss stabilizes AURKA through inhibiting FBXW7-mediated degradation

**Fig. 6:**
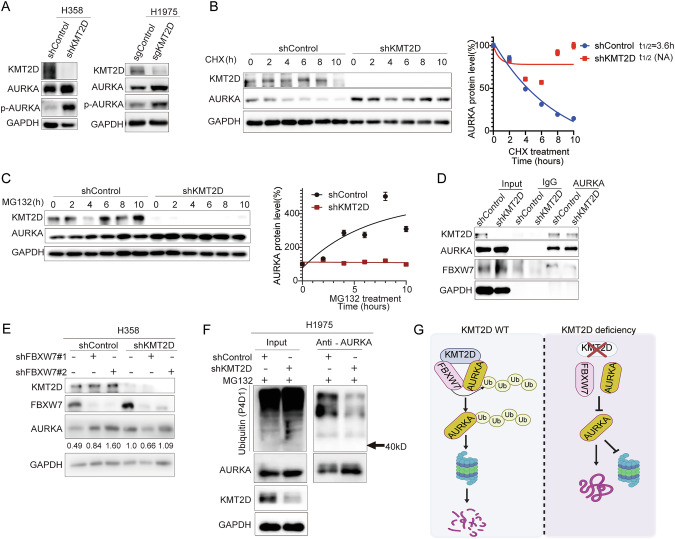
KMT2D loss stabilizes AURKA through inhibiting FBXW7-mediated degradation. **A** Western blot analysis of H358 and H1975 cells with stable KMT2D knockdown (shKMT2D) or knockout (sgKMT2D), compared to corresponding control cells (shControl or sgControl). GAPDH served as a loading control. **B** Left: Western blot analysis of AURKA protein levels in H358 shControl and shKMT2D cells treated with cycloheximide (CHX, 50 μg/mL) for the indicated times (0–10 h). GAPDH was used as a loading control. Right: Quantification of AURKA protein levels over time, normalized to time 0. Quantification of AURKA protein levels over time, normalized to time 0. The half-life (t₁/₂) of AURKA was calculated using a one-phase decay model. **C** Left: Western blot analysis of AURKA levels in H358 shControl and shKMT2D cells treated with the proteasome inhibitor MG132 (10 µM) for the indicated durations (0–10 h). GAPDH served as a loading control. Right: Quantification of AURKA protein levels normalized to time 0. **D** Co-immunoprecipitation (Co-IP) was performed in H358 shControl and shKMT2D cells using an anti-AURKA antibody or IgG control, followed by immunoblotting for KMT2D, AURKA, and FBXW7. Input lysates and IgG pulldowns served as controls. GAPDH was used as a loading control. **E** Western blot analysis of AURKA, FBXW7, and KMT2D protein levels in H358 shControl and shKMT2D cells transduced with two independent shRNAs targeting FBXW7 (shFBXW7#1 and #2). Densitometric quantification of AURKA relative to GAPDH is shown below each lane. **F** shControl and shKMT2D cells were treated with MG132 and subjected to immunoprecipitation with anti-AURKA antibody. Immunoprecipitates were immunoblotted with P4D1 (anti-ubiquitin) to detect ubiquitinated AURKA. Whole-cell lysates (WCL) verified efficient KMT2D depletion in shKMT2D cells, GAPDH served as a loading control. **G** Schematic illustration the role of KMT2D in regulating AURKA stability (Created with BioRender.com).

We next investigated the mechanistic basis of this dependency. Given AURKA’s critical role in mitotic progression, we hypothesized that KMT2D loss induces a state of mitotic reliance to support the proliferative and lineage plasticity of squamous-like tumor cells. Immunoblot analysis revealed that KMT2D-deficient cells exhibited markedly elevated levels of total and phosphorylated AURKA compared to wild-type controls (Figs. 6A and S6A; Original data file 1). This increase was accompanied by enhanced proliferative activity (Fig. S6B), suggesting that AURKA hyperactivation may contribute to the mitotic dependency imposed by KMT2D loss. Surprisingly, quantitative PCR analysis showed no significant difference in AURKA mRNA levels between wild-type and KMT2D-deficient cells (Fig. S6C), indicating that the observed protein upregulation is likely due to post-translational regulation. To determine whether KMT2D modulates AURKA stability, we performed cycloheximide (CHX) chase assays to assess AURKA degradation dynamics. In wild-type cells, AURKA levels declined progressively over time, consistent with proteasome-mediated turnover. In contrast, AURKA remained relatively stable in KMT2D-deficient cells, indicating impaired degradation (Fig. 6B; Original data file 1). We further treated cells with the proteasome inhibitor MG132. In wild-type cells, MG132 induced time-dependent accumulation of AURKA, whereas in KMT2D-deficient cells, AURKA levels showed minimal change, suggesting that AURKA is already stabilized in the absence of KMT2D (Fig. 6C; Original data file 1). To investigate how KMT2D loss affects AURKA stability, we searched the BioGRID interaction database for proteins that interact with both KMT2D and AURKA (Supplementary Table S5). This analysis identified a small number of shared interactors, among which FBXW7, a well-characterized E3 ubiquitin ligase responsible for AURKA degradation during mitotic exit [56], emerged as a key candidate (Fig. S6D). Co-immunoprecipitation assays further confirmed that FBXW7 interacts with both KMT2D and AURKA (Fig. S6E; Original data file 1). Given its functional relevance, we hypothesized that KMT2D may regulate AURKA turnover by modulating its association with FBXW7. Supporting this hypothesis, Co-IP experiments revealed a robust interaction between AURKA and FBXW7 in wild-type cells, which was markedly reduced in KMT2D-deficient cells (Figs. 6D and S6F; Original data file 1). Functionally, FBXW7 knockdown increased AURKA protein in shControl cells, whereas this effect was markedly attenuated in KMT2D-knockdown cells (Fig. 6E; Original data file 1). In line with these findings, pharmacogenomic analysis of the Genomics of Drug Sensitivity in Cancer dataset revealed that FBXW7-mutant cancer cell lines display significantly greater sensitivity to the AURKA inhibitor alisertib compared with FBXW7-wild-type counterparts (Fig. S6G). Together, these results indicate that KMT2D is required for efficient FBXW7-mediated degradation of AURKA, likely by facilitating the formation or stabilization of the AURKA-FBXW7 complex. Furthermore, immunoprecipitation of AURKA followed by ubiquitin blotting revealed a marked reduction in AURKA ubiquitination upon KMT2D knockdown, further supporting a role for KMT2D in promoting FBXW7-dependent ubiquitin-mediated turnover of AURKA (Fig. 6F; Original data file 1).

Collectively, these findings demonstrate that KMT2D loss impairs FBXW7-mediated ubiquitination of AURKA, resulting in its stabilization and hyperactivation (Fig. 6G). This mechanism likely underlies the mitotic addiction and AURKA inhibitor sensitivity observed in KMT2D-deficient tumors.

### AURKA inhibition impairs squamous identity and overcomes TKI resistance in NSCLC

**Fig. 7:**
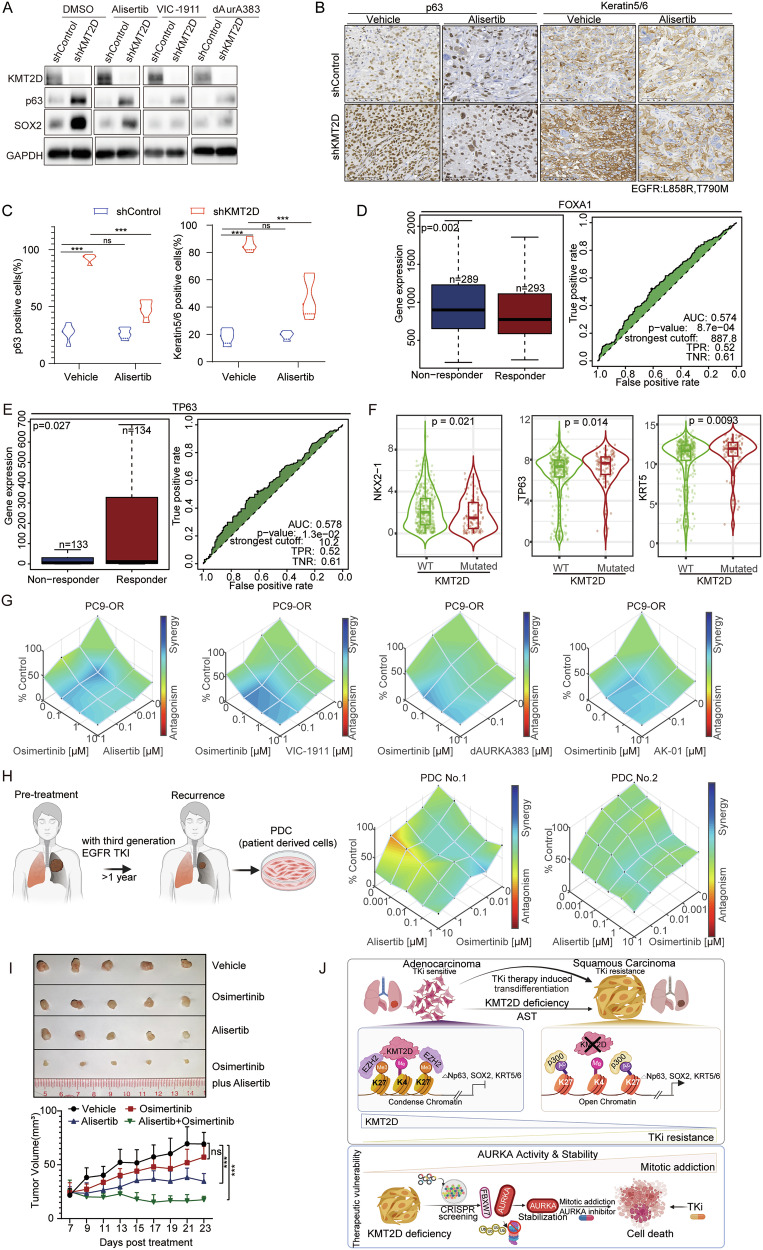
AURKA inhibition impairs squamous identity and overcomes TKI resistance in NSCLC. **A** Western blot analysis of squamous lineage-related protein expression in EGFR mutant (H1975) lung cancer cells, with and without KMT2D knockdown, following 72 h treatment with AURKA inhibitors. **B** Immunohistochemical (IHC) analysis of p63 (left panels) and Keratin 5/6 (right panels) expression in xenograft tumor sections derived from either control shRNA (shControl) or shRNA targeting KMT2D (shKMT2D) NSCLC cells with EGFR mutation (L858R + T790M), treated with vehicle or the AURKA inhibitor Alisertib. Representative images illustrate p63-positive nuclei or Keratin 5/6-positive cytoplasmic staining (brown). Scale bars: 100 µm. **C** The box plots quantify the percentage of p63 or Keratin 5/6 positive cells under each condition. Data are mean ± SD (n = 4, 5; 5–10 random HPFs per tumor averaged), using two-way ANOVA, ***P < 0.001; ns not significant. **D**, **E** Boxplot comparing the expression of FOXA1 and TP63 between non-responders (blue) and responders (red) to the AURKA inhibitor alisertib. Receiver operating characteristic (ROC) curve evaluating the predictive power of FOXA1 and TP63 expression in distinguishing responders from non-responders. Data obtained from solid tumor cells. *P* value is indicated. **F** Analysis the log₂ expression of adenocarcinoma and squamous lineage markers in KMT2D wild-type (WT) versus KMT2D-mutated samples across non-small cell lung cancer (NSCLC). The data obtained from TCGA and analyzed by TIMER. **G** Representative Combenefit synergy surface for osimertinib-resistant PC9 (PC9-OR) cells treated for 72 h with osimertinib and AURKA inhibitors dose matrix. Synergy was analyzed in Combenefit (Loewe model), blue indicates synergy and red antagonism. Axes show drug concentrations (μM) (n = 3). **H** Left: Schematic of sample derivation: tumor resection at recurrence after >1 year on third-generation EGFR-TKI, followed by establishment of patient-derived cells (PDCs) (Created with BioRender.com). Right (PDC No.1 and PDC No.2): Combenefit 3D dose response synergy surfaces showing PDC viability (% control) after 72 h co-treatment with alisertib and osimertinib. Synergy was assessed using the Loewe model (n = 3). **I** Top: Representative tumors after 23 days of treatment with vehicle, osimertinib (3.5 mg/kg), alisertib (30 mg/kg), or the combination. Bottom: Tumor volume over time under the indicated treatments (black, vehicle; red, osimertinib; blue, alisertib; green, combo). Data are mean ± SD (n = 5), two-way ANOVA. ns not significant; ***P < 0.001. **J** Schematic summary of this study (Created with BioRender.com).

Given that AURKA is stabilized and hyperactivated in KMT2D-deficient cells, we next asked whether this activity is not only a byproduct of KMT2D loss but functionally required to sustain the squamous-like cell state and associated therapy resistance. To determine whether AURKA activity is required to maintain the squamous program in KMT2D-deficient cells, we treated cells with multiple AURKA-targeting agents, including selective inhibitors (alisertib, VIC-1911, AK-01) and a targeted degrader (dAurK383). These treatments consistently reduced the expression of key squamous lineage regulators p63 (ΔNp63) and SOX2 specifically in KMT2D-deficient cells (Figs. 7A and S7A; Original data file 1), suggesting that AURKA activity is essential for maintaining the squamous-like phenotype induced by KMT2D loss. Supporting these in vitro findings, immunohistochemical analysis of xenograft tumors harboring EGFR or KRAS mutations showed strong expression of p63(ΔNp63) and keratin 5/6 in vehicle-treated KMT2D-deficient tumors, which was markedly suppressed following alisertib treatment (Figs. 7B, C and S7B). These results collectively demonstrate that KMT2D-deficient tumors depend on AURKA signaling to sustain their squamous identity and that AURKA inhibition impairs this lineage state.

To establish the clinical relevance of KMT2D deficiency, we examined whether squamous lineage markers could stratify responses to the AURKA inhibitor alisertib. In solid tumor cohorts treated with alisertib, non-responders displayed significantly higher expression of the adenocarcinoma-associated factor *FOXA1*, whereas responders were enriched for squamous markers such as *TP63*, *KRT5*, and *KRT6B* (Figs. 7D, E and S7C). These results suggest that KMT2D deficiency-driven squamous differentiation predicts enhanced sensitivity to AURKA inhibition, whereas tumors retaining FOXA1 expression may be less responsive.

We next assessed the relationship between KMT2D alterations and lineage identity using transcriptomic data from The Cancer Genome Atlas (TCGA). In NSCLC, KMT2D mutations correlated with reduced expression of the adenocarcinoma-specific transcription factor *NKX2-1* and increased expression of squamous lineage factors, including *TP63*, *KRT5*, *KRT14*, and *KRT16* (Figs. 7F and S7D). These observations reinforce our earlier findings that KMT2D loss drives adenocarcinoma-to-squamous transition (AST) and promotes acquired resistance to tyrosine kinase inhibitors (TKIs). Given the ongoing clinical development of AURKA inhibitors (e.g., alisertib, VIC-1911) for osimertinib-resistant EGFR-mutant NSCLC [57, 58], we tested whether co-targeting AURKA and EGFR could overcome resistance. In PC9-OR cells and patient-derived cancer cells (PDCs), combination treatments pairing osimertinib with alisertib, VIC-1911, dAURKA383, or AK-01 produced synergistic effects, as quantified by synergy scoring and visualized by blue regions in the interaction landscapes (Fig. 7G). Notably, patient-derived cancer cells (PDC No.1 and No.2) also exhibited significant synergy when treated with a combination of osimertinib and either alisertib or VIC-1911 (Figs. 7H and S7E). Moreover, the combination of osimertinib and alisertib in osimertinib-resistant lung cancer model significantly inhibited AKT activation, increased cleaved Caspase-3 levels and reduced tumor size (Figs. 7I and S7F, G; Original data file 1), highlighting that co-targeting AURKA and EGFR may offer a promising therapeutic strategy to overcome osimertinib resistance in lung cancer.

Together, this study demonstrates that KMT2D deficiency in NSCLC promotes adeno to a squamous transition through epigenetic reprogramming. Specifically, loss of KMT2D disrupts the equilibrium between H3K27 methylation and acetylation, with compensatory p300 activity promoting upregulation of squamous lineage markers including ΔNp63, SOX2, KRT5, and KRT6A. This reprogramming not only promotes squamous transition but also confers resistance to TKIs and alters mitotic signaling, resulting in a dependency on the activity of mitotic kinase AURKA (mitotic addiction). Mechanistically, KMT2D loss impairs the interaction between AURKA and the E3 ubiquitin ligase FBXW7, thereby attenuating FBXW7-mediated ubiquitination and degradation of AURKA and leading to its pathological stabilization. Therapeutic strategies targeting AURKA with inhibitors (e.g., alisertib, VIC-1911) in combination with osimertinib demonstrated synergistic effects, particularly in resistant models, suggesting AURKA addiction as a critical vulnerability (Fig. 7J). These findings reveal the interplay between epigenetic alterations, lineage plasticity, and mitotic signaling, providing a strong rationale for combination therapies to overcome TKI resistance in KMT2D-deficient lung cancers.

## Discussion

Lineage infidelity is a recognized route of tumor adaptation across cancers [59, 60]. AST occurs particularly in the context of treatment resistance and tumor evolution [10, 61, 62], consistent with studies showing that lineage plasticity and cell-of-origin programs shape tumor adaptation and heterogeneity in lung cancer [63, 64]. Clinically, this histologic shift is associated with poor outcomes and reduced responsiveness to adenocarcinoma-directed regimens. Here we show that KMT2D deficiency promotes AST, with chromatin reprogramming, attenuation of the adenocarcinoma program (NKX2-1/TTF-1, FOXA1), and induction of squamous lineage factors (ΔNp63/p63, SOX2, KRT5). Functionally, KMT2D loss reduces EGFR-TKI sensitivity, supporting a model in which loss of enhancer control destabilizes LUAD identity and facilitates adaptive lineage switching.

KMT2D catalyzes H3K4me1 at enhancers and promotes H3K27ac through recruitment of CBP/p300 [65], and its functional interplay with PRC2/EZH2-mediated H3K27me3 suggests coordinated regulation of activation and repression [52]. In keratinocytes, KMT2D cooperates with p63 to sustain epidermal enhancers [66, 67], whereas in LUAD, which lacks a pre-existing p63 network, KMT2D loss instead perturbs adenocarcinoma-specific enhancer architecture, creating a permissive chromatin state that facilitates induction of p63(ΔNp63) and other squamous factors. Consistent with this, we observe p300 engagement at ΔNp63-associated elements in the setting of KMT2D loss, compatible with a co-activator shift that supports squamous programs. KMT2D is among the most frequently altered epigenetic regulators in NSCLC [34, 68]. When mutated, KMT2D’s diminished enzymatic activity compromises its tumor suppressor function, leading to widespread epigenomic reprogramming and clonal expansion, as observed in lung cancer[69, 70], head and neck squamous cell carcinoma (HNSCC) [71, 72], cutaneous squamous cell carcinoma (cSCC) [69], and melanoma [73]. Unlike canonical tumor suppressors that undergo biallelic inactivation, KMT2D frequently exhibits monoallelic loss, supporting a dosage-sensitive, haplo-insufficient mode of action in cancer [74]. Notably, KMT2D inactivation has been linked to enhanced glycolytic activity in KRAS-driven tumors and the induction of squamous differentiation in lung basal cell organoids [15, 70]. Similar chromatin-based lineage shifts have been reported in triple-negative breast cancer, urothelial carcinoma, and prostate cancer, where loss of KMT2D or its paralog KMT2C is associated with enhanced metastatic potential or therapy-induced differentiated phenotype [75–77]. A comparable mechanism has been described in pancreatic adenocarcinoma, where loss of KDM6A (UTX), a COMPASS-like partner of KMT2D, activates ΔNp63-driven super-enhancers to induce a squamous-like phenotype [25]. Concordantly, our findings extend this lineage logic to a glandular context, where KMT2D loss shifts cell state toward a squamous program. The specific outcome likely depends on the resident transcription-factor circuitry and baseline chromatin accessibility, producing convergent squamous phenotypes through lineage-selective mechanisms.

This established role of KMT2D loss in driving lineage plasticity prompted us to investigate its relevance in clinical resistance. Supporting this premise, prior genomic profiling of osimertinib-resistant NSCLC reveals recurrent KMT2D alterations, and single-cell data implicate these mutations in clonal evolution [78, 79]. Building on this clinical signal, we identify the mitotic kinase AURKA as a selective vulnerability in KMT2D-deficient states. Our in vitro systems, organoids, patient-derived cells, and orthotopic xenografts demonstrate that AURKA inhibition constrains tumor growth in KMT2D-deficient models, providing a compelling rationale for patient stratification based on KMT2D status. The mechanistic basis for this dependency lies in the regulation of AURKA stability. While FBXW7 is known to ubiquitinate and target AURKA for proteasomal degradation [56, 80], and has been reported to intersect with KMT2D-dependent pathways [81]. KMT2D deficiency disrupts the interaction between AURKA and FBXW7, impairing ubiquitination and degradation of AURKA, and leading to its pathological stabilization. This mechanistic insight explains how KMT2D loss couples epigenetic reprogramming to mitotic addiction. It also suggests that defects in the KMT2D-FBXW7 axis may broadly contribute to lineage plasticity and therapeutic resistance in NSCLC. Collectively, our study establishes AURKA inhibition as an effective approach to exploit the vulnerabilities conferred by KMT2D loss, offering a therapeutic window to overcome TKI resistance. The documented preliminary efficacy of AURKA inhibitors like alisertib in early-phase trials [57, 58] further supports the translational potential of combining these agents with TKIs in KMT2D-altered, TKI-resistant NSCLC.

While our data identify KMT2D as an intrinsic chromatin regulator that orchestrates AST, we acknowledge that AST is a multifactorial process shaped by both cell-intrinsic (e.g., additional epigenetic regulators and signaling pathways) and microenvironmental influences [5, 6, 19]. Extrinsic factors within the tumor microenvironment, such as stromal interactions, immune modulation, and metabolic cues, likely amplify AST and drug resistance [82–84], warranting further investigation. Importantly, it is essential to discriminate bona fide trans-differentiation from therapy-driven selection or expansion of pre-existing adeno-squamous or squamous subclones, this question necessitates genetically barcoded or lineage-traced models. To address these gaps, future work should employ inducible Kmt2d genetically engineered mouse models (GEMMs) coupled with lineage tracing and spatial transcriptomics to define clonal dynamics, tumor ecology, and resistance mechanisms with temporal and spatial precision. Furthermore, to establish KMT2D expression as a reliable predictor of EGFR-TKI response, future research should leverage larger, diverse clinical cohorts across multiple institutions. Prospective, well-powered studies are essential to confirm KMT2D’s role as a biomarker and to rigorously assess the therapeutic potential of combining AURKA inhibitors with EGFR-targeted therapies. By integrating advanced genomic profiling and real-world evidence, these efforts will drive the development of personalized treatment paradigms, enabling tailored therapies that address resistance mechanisms and improve long-term patient outcomes.

## Materials and methods

### Cell lines and culture conditions

Human non-small cell lung cancer (NSCLC) cell lines, including H358, HCC44, H1975, H1975_OR, SW1573, DMS114, and H2170 were purchased from the American Type Culture Collection (ATCC, Manassas, VA, USA). These cells were cultured in RPMI-1640 medium (Gibco, Thermo Fisher Scientific, Waltham, MA, USA), supplemented with 10% (v/v) fetal bovine serum (FBS, Gibco, Grand Island, NY, USA) and 1% penicillin-streptomycin. HEK293T cells, as well as PC9 parental (PC9_Par) and osimertinib-resistant PC9 (PC9_OR) sublines, were maintained in Dulbecco’s Modified Eagle Medium (DMEM, Gibco) with 10% FBS. All cell cultures were incubated under standard conditions at 37 °C in a humidified atmosphere containing 5% CO_2_. Cell line identity was routinely authenticated by short tandem repeat (STR) profiling, and all cultures were regularly screened for mycoplasma contamination using PCR-based methods to ensure experimental integrity. Plasmid transfection in HEK293T cells was conducted using polyethyleneimine (PEI, Sigma-Aldrich, St. Louis, MO, USA), following the manufacturer’s recommendations for reagent preparation and cell handling. Lentiviral particles were generated for stable cell line creation by transfecting HEK293T cells with lentiviral plasmids, as previously described [53]. Virus-containing supernatants were collected, filtered, and applied to target cells in the presence of 2 µg/mL Polybrene (Cat# 107689; Sigma Aldrich) to enhance transduction efficiency. After 48 h, selection of transduced cells was initiated using the appropriate antibiotic, yielding stable cell lines for downstream experimentation.

### Immunoprecipitation and western blotting

For co-immunoprecipitation (co-IP) assays, cells were lysed in NETN buffer (20 mM Tris-HCl, pH 8.0; 100 mM NaCl; 0.5% NP-40; and 1 mM EDTA) supplemented with protease inhibitors and phosphatase inhibitors on ice for 30 min. Cell lysates were pre-cleared with Protein A/G agarose beads (Cat# HY-K0202, MedChem Express) for 1 h at 4 °C and then incubated overnight at 4 °C with the indicated primary antibodies or control IgG (Supplementary Table S6). Immune complexes were captured with Protein A/G beads for 2–4 h, washed extensively with lysis buffer, and eluted by boiling in SDS loading buffer. For standard Western blotting, whole-cell lysates were prepared using the same RIPA buffer supplemented with protease and phosphatase inhibitors. Equal amounts of protein from either input lysates or co-IP samples were resolved by SDS-PAGE and transferred to polyvinylidene difluoride (PVDF) membranes (Merck Millipore). Membranes were blocked with 5% bovine serum albumin (BSA) and probed with the appropriate primary antibodies (Supplementary Table S6) at the recommended dilutions. After incubation with HRP-conjugated secondary antibodies, including anti-Mouse (Cat# 31430, Thermo Fisher Scientific) or anti-Rabbit (Cat# 31460, Thermo Fisher Scientific), signals were detected using an enhanced chemiluminescence (ECL) imaging system (Cat# SQ201L, EpiZym).

### Immunofluorescence and confocal microscopy

Cells were seeded onto coverslips and incubated for 24 h. Cells and organoids were fixed with 4% paraformaldehyde (PFA) for 10 min at room temperature, followed by permeabilization with 0.5% Triton X-100 in PBS for 5 min. After three washes with 0.005% Triton X-100 in PBS, primary antibody staining was performed by incubating the cells overnight at 4 °C with anti-AURKA (Cat# D3E4Q, Cell Signaling Technology), anti-p63(Cat# AF1993, Beyotime), anti-ΔNp63 (Cat# 67825, Cell Signaling Technology), anti-Keratin 5/6 (Cat# AG2422, Beyotime), and Cleaved Caspase-3 (Asp175) (Cat# 9661, Cell Signaling Technology) antibodies, diluted 1:100 in PBS containing 1% BSA. After washing with 0.005% Triton X-100 in PBS, the cells were incubated with Alexa Fluor-conjugated secondary antibodies (Alexa Fluor 488 or Alexa Fluor 594, Life Technologies) diluted 1:200 in PBS for 2 h at room temperature. Following additional washes, the cells were stained with DAPI (1 µg/ml; CAS# D954, Sigma) to visualize nuclei. Images were captured using a Zeiss LSM 880 laser scanning confocal microscope equipped with oil immersion objective.

### CRISPR/Cas9 kinome library construction and screening

A CRISPR/Cas9 kinome library was constructed as previously described [53, 85]. Briefly, HEK293T cells were co-transfected with the CRISPR-kinome library plasmids, the vesicular stomatitis virus glycoprotein plasmid (pMD2.G, Addgene, Watertown, MA, USA), and the packaging plasmid (psPAX2, Addgene), using polyethyleneimine (Cat#408727, Sigma-Aldrich). Fresh DMEM was added 12 h post-transfection to enhance viral production. Lung cancer cells with KMT2D deficiency were transduced with the viral supernatant at a multiplicity of infection (MOI) of 0.3 for 24 h, followed by selection with 2 µg/mL puromycin (MP Biomedicals) for 7 days. Transduced cells were expanded and collected on days 7 and 14 for genomic DNA extraction. The details are listed in Supplementary Table S4.

### CRISPR/Cas9 epigenetic library screen and sequencing

We performed a high-throughput CRISPR/Cas9 screen targeting epigenetic regulators, as previously described [31]. Briefly, the lentiviral library was packaged in HEK293T cells, filtered through a 0.45 µm membrane, and used to infect lung cancer cells at a multiplicity of infection (MOI) of 0.3. Transduced cells underwent puromycin selection for 7 days to ensure stable sgRNA integration. Post-selection, cells were split into three treatment groups: vehicle control (DMSO), alisertib (100 nM), and VIC- 1911 (150 nM). Samples were collected at 7 days post-treatment, maintaining a minimum of 2 × 10⁷ cells per group to ensure at least 300-fold library coverage. Genomic DNA was extracted using the HP Tissue DNA Midi Kit (Omega Bio-Tek). Amplification of sgRNA sequences for sequencing library preparation was performed via a two-round PCR protocol. Libraries were sequenced on Illumina NovaSeq platforms (150 bp, paired-end). Data analysis was conducted with MAGeCK-MLE (v0.5.9.5) [86], using beta scores to estimate gene essentiality under each treatment condition. The gene essentiality estimation *β* scores are provided in Supplementary Table S4.

### Plasmid construction

To achieve gene knockdown, doxycycline (Dox)-inducible shRNAs targeting KMT2D were cloned into the TET-ON lentiviral vector (Tet-pLKO-Puro, Addgene #21915). Non-targeting shRNA (pLKO-Tet-On-shRNA-Control) served as a negative control. Stable cell lines were established by puromycin selection for 7 days, and shRNA expression was induced by adding 2 µg/ml Dox to the culture medium. For gene knockout, guide RNAs (gRNAs) targeting KMT2D were designed using the CHOPCHOP online tool [87] and cloned into the lentiCRISPR v2 vector (Addgene #52961). The efficiency of knockdown or knockout was validated by Western blot analysis. All plasmid constructs were verified by Sanger sequencing. Primer sequences are provided in Supplementary Table S6.

### Cell viability and clonogenic assays

NSCLC cell lines were treated with serial drug dilutions for 72 h, and viability was measured using the CCK-8 kit (Cat# 40203ES88, Yeasen). IC₅₀ values were calculated by log (inhibitor) vs. normalized response fitting. For clonogenic assays, H358, H1975, and HCC44 stably expressing shControl or shKMT2D were seeded at a density of 500 cells per well in 6-well plates. Cells were treated with various concentrations of AURKA inhibitors, including alisertib, AK-01, dAurK383, and VIC-1911. Following 10–14 days of incubation, cell colonies were fixed with 70% ethanol, stained with 0.5% crystal violet solution, and imaged. Colony formation assays were conducted in biological triplicates to ensure reproducibility. Wells/plates were excluded only by pre-specified QC criteria: contamination, edge-effect artifacts, absorbance outside the linear range, or non-convergent/poor dose-response fits; affected assays were repeated.

### Clinical specimen study

Human lung cancer tissues used for organoid and PDC establishment were collected under approval from the Institutional Ethics Committee of Sun Yat-sen University Cancer Center (approval No. G2024-028-01). Written informed consent was obtained from all patients or their legal guardians prior to tissue collection, in accordance with the institutional ethical guidelines. The sample information was listed in Supplementary Table S6.

### Patient-derived organoids

Patient-derived organoids (PDOs) were established from resected tumor samples obtained from non-small cell lung cancer (NSCLC) patients. All patients provided informed consent, and the study was approved by the Ethics Committee of Sun-Yat-Sen University Cancer Center (approval no. G2024-028-01). Tumor specimens were mechanically dissociated into small fragments using sterile scalpels, followed by enzymatic digestion with collagenase (Type IV, Gibco) for 30 min at 37 °C, based on published protocols with some modifications to optimize dissociation and viability [88]. After digestion, cells were washed, pelleted by centrifugation, and resuspended in Matrigel (Cat# 356231, Corning). Organoids were cultured by embedding cells in Matrigel, which was plated in 24-well plates and overlaid with organoid culture medium consisting of Advanced DMEM/F12 (Gibco), supplemented with B27 (Gibco), N2 (Gibco), epidermal growth factor (EGF, 50 ng/mL; PeproTech), Noggin (100 ng/mL; PeproTech), and antibiotics (penicillin-streptomycin, Gibco). After organoid formation, PDOs were treated for 7 days with DMSO (vehicle) or with alisertib (100 nM), VIC-1911 (200 nM), osimertinib (100 nM), or osimertinib (100 nM) + alisertib (100 nM). Medium and inhibitors were refreshed every 3 days. Organoids were imaged using bright-field microscopy at the end of the treatment period to evaluate morphological alterations. Quantitative analysis, including organoid size and number, was performed using ImageJ software. All experiments were performed in three independent biological replicates to ensure reproducibility.

### Flow cytometry analysis of apoptosis

Lung cancer cell lines (H358, H1975, SW1573, DMS114, and H2170) were stably infected with lentivirus expressing either shControl or shKMT2D. Cells were treated with DMSO (vehicle control), alisertib, or VIC-1911 at the indicated concentrations for 72 h. After treatment, cells were harvested by trypsinization, washed twice with ice-cold phosphate-buffered saline (PBS), and stained using the Annexin V Apoptosis Detection Kit (Cat#AP006, ES Science). Specifically, cells were resuspended in binding buffer and incubated with Annexin V-APC and propidium iodide (PI) for 30 min in the dark, following the manufacturer’s instructions. Following staining, apoptotic and non-apoptotic cells were analyzed by flow cytometry using a BD FACSCanto II (BD Biosciences). A minimum of 10,000 events were collected per sample. Flow cytometry data were analyzed using FlowJo software (BD Biosciences) to quantify the percentage of cells in early apoptosis (Annexin V positive, PI negative) and late apoptosis/necrosis (Annexin V positive, PI positive). All experiments were conducted in three independent biological replicates, and data were statistically analyzed to assess treatment effect.

### In vivo xenograft model

All animal studies complied with institutional and national ethical regulations for laboratory animal welfare. All procedures were approved by the Sun Yat-sen University Cancer Center IACUC/Animal Ethics Committee (protocol [SYUCC-IACUC-L102032021070B]). Mice were housed under SPF conditions with environmental enrichment and ad libitum food and water. Humane endpoints were pre-specified (ulceration, >20% body-weight loss, severe morbidity, or maximum tumor burden [e.g., 1500 mm³]), and animals meeting endpoints were humanely euthanized (isoflurane anesthesia followed by CO_2_ overdose). Animals were randomized when tumors reached ~100 mm³, with baseline volumes balanced across groups. The mouse was the biological unit; inclusion/exclusion criteria were defined a priori.

H358 and H1975 NSCLC cells stably expressing shControl or shKMT2D (1 × 10⁶ cells in PBS/Matrigel) were injected subcutaneously into the flanks of 6-week-old female nude mice (n = 4, 5 per group). Once tumor volumes reached approximately 100 mm^3^, mice were randomized into treatment groups receiving either vehicle control or the AURKA inhibitor alisertib (30 mg/kg) administered daily via intraperitoneal injection for 21 or 25 days. Tumor volumes were measured every 2 days using digital calipers, with volume calculated according to the formula: (length × width^2^)/2. Mice were monitored for any signs of distress or toxicity during the treatment period. At endpoint, mice were euthanized as above, tumors were excised, weighed, and processed for H&E and IHC (adenocarcinoma, squamous, proliferation, apoptosis markers). Tumor-growth curves and final weights were analyzed as specified in the statistics section. For PC9 osimertinib-resistant (PC9-OR) models, 1 × 10⁶ cells in 100 µL Matrigel:PBS (1:1) were injected subcutaneously into 6-week-old NOD-SCID male mice (n = 5 per group). The mice were treated daily starting on day 7 through day 23 with either vehicle control, osimertinib (3.5 mg/kg/day), alisertib (30 mg/kg/day), or a combination of osimertinib (3.5 mg/kg/day) and alisertib (30 mg/kg/day). Tumor volumes were recorded at every 2 days post-treatment and volume was calculated using the same formula. Animals were euthanized at the endpoint and tumors were processed for histology. All exclusions and humane-endpoint events were recorded and reported, the exact number of mice per group is provided in the figure legends.

### Orthotopic injection in immunocompetent mouse model

Single cells derived from genetically engineered *Kras*^*G12D*^*; Trp53*^*L/L*^*; Myc* organoids, with or without *Kmt2d*(*Mll4*) knockout, were prepared for orthotopic injection as previously reported [89]. Briefly, a total of 2 × 10^5^ cells per mouse were injected into the left lung lobes of C57BL/6 immunocompetent mice under anesthesia. On day 7, mice were randomized, stratified by baseline bioluminescence (BLI), to vehicle or alisertib (30 mg/kg, intraperitoneal, once daily on days 7–28) using a random-number table. Tumor burden was monitored longitudinally by BLI. Pre-specified exclusion criteria were failed/ectopic injection (no BLI signal), peri-operative mortality, severe respiratory distress, >20% body-weight loss, or reaching humane endpoints, affected animals were euthanized and recorded. Tumor growth was monitored longitudinally using bioluminescence imaging (BLI) following the administration of D-luciferin substrate, allowing non-invasive tracking of tumor progression over time. At the endpoint, lungs were harvested for H&E and IHC. All procedures were approved by the Sun Yat-sen University Cancer Center IACUC/Animal Ethics Committee (protocol [SYUCC-IACUC-L102032021070B]), inclusion/exclusion criteria were defined a priori.

### Histological and immunohistochemical analysis

NSCLC tissue specimens, adjacent normal lung tissues, and excised tumors from both xenograft and orthotopic models were fixed in 10% neutral buffered formalin for 24 h. After fixation, tissues were processed, embedded in paraffin, and sectioned at a thickness of 4 µm. Hematoxylin and Eosin (H&E) staining was performed on deparaffinized sections to assess general tumor morphology. For IHC analysis, tissue sections were deparaffinized, rehydrated, and subjected to antigen retrieval by heating the sections in citrate buffer (pH 6.0). Endogenous peroxidase activity was blocked by incubation in 0.3% hydrogen peroxide (H₂O₂) according to the manufacturer’s instructions. The sections were then incubated with the following primary antibodies at 4 °C overnight: KMT2D (Cat#27266-1-AP, Proteintech), p63 (Cat#A1993, Beyotime), p63 (Cat#13109, CST), deltaNp63 (Cat# 67825, CST), p40 (Cat#RMA-0815, MXB), TTF-1 (Cat#ab133638, Abcam), KRT5/6 (Cat#AG2422, Beyotime), Ki67 (Cat#12202, CST), and Cleaved PARP-1 (Cat#AG1043, Beyotime). Following primary antibody incubation, sections were washed and incubated with secondary antibodies according to the manufacturer’s protocol. The tissue samples underwent immunohistochemical staining using 3,3’-diaminobenzidine (DAB) as the chromogen to detect target antigens, followed by hematoxylin counterstaining to visualize cell nuclei. Post-staining, the sections were digitized with a KFBIO Digital Pathology Slide Scanner. Quantitative analysis was conducted using ImageJ software, focusing on staining intensity and the proportion of positive cells. For each marker, five randomly selected fields per sample were evaluated. The results were reported as the percentage of positively stained cells relative to the total cell count in each tumor slides.

### ATAC sequencing

The ATAC (Assay for Transposase-Accessible Chromatin) method was applied based on the published protocol[90, 91]. The Doxycycline-induced shControl and shKMT2D cells were plated at 5 × 10^5^ cells onto 10 cm plates and harvested after 3 days of induction by Doxycycline. 50,000 cells per sample were resuspended in 1 mL of cold PBS. Cells were centrifuged at 500 × *g* for 5 min in a pre-chilled (4 °C) fixed-angle centrifuge. After centrifugation, the supernatant was carefully and completely aspirated. Cell pellets were then resuspended in 50 μL of ATAC-seq RSB containing 10 mM Tris-HCl pH 7.4, 10 mM NaCl, 3 mM MgCl2, 0.1% NP40, 0.1% Tween-20, and 0.01% digitonin by pipetting up and down three times. This cell lysis reaction was incubated on ice for 3 min. After lysis, 1 mL of ATAC-seq RSB containing 0.1% Tween-20 (without NP40 or digitonin) was added, and the tubes were inverted to mix. Nuclei were then centrifuged for 5 min at 500 × *g* in a pre-chilled (4 °C) fixed-angle centrifuge. The supernatant was removed, and the nuclei were resuspended in 50 μL of transposition reaction mix using the TruePrep DNA Library Prep Kit V2 for Illumina, which includes 5× TTBL and TTE Mix V50 (Cat#TD711-01, Vazyme) to construct ATAC-seq libraries. Transposition reactions were incubated at 37 °C for 30 min in a thermomixer with shaking at 1000 r.p.m. Reactions were cleaned up with the MinElute Reaction Cleanup Kit (Qiagen) to purify transposed DNA. Quality control of the tagmented DNA was performed using a TapeStation. Libraries were amplified using the TruePrep DNA Index Kit V2 for Illumina sequencing (Vazyme) and the NEBNext high-fidelity PCR mix. The libraries were sent to Novogene for sequencing with the NovaSeq platform (150 bp, paired-end).

### ChIP-qPCR

shControl and shKMT2D cells were plated at 5 × 10^5^ cells into 10 cm plates, and were induced by Doxycycline. The procedure was followed as previously described with some modifications [92]. Briefly, cells were washed in phosphate-buffered saline (PBS) and crosslinked with 1% Formaldehyde for 10 min. Crosslinked cell lines were quenched with 125 mM glycine for 5 min at room temperature. After quenching, cells were hypotonically lysed, and sonicated in a Covaris E220 sonicator in 0.1% SDS/TE. Soluble chromatin was immunoprecipitated with 2 μg of H3K4me1, H3K27ac, H3K27me3, and IgG antibody. The antibodies were retrieved with ChIP-grade ProteinA/G Magnetic Beads (Cat#26162, Thermo Fisher), and bound chromatin DNA was purified using a QIAquick PCR Purification Kit (Qiagen). Quantitative PCR was performed using SYBR Green Master Mix (Vazyme) on a BioRad Real-Time PCR system. Enrichment was calculated as a percentage of input DNA or normalized to control IgG. Primer sequences targeting enhancer or promoter regions are provided in Supplementary Table S6.

### CUT-tag sequencing

The CUT&Tag assay was performed using Hieff NGS® In-Situ DNA Binding Profiling Library Prep Kit for Illumina® V2 CUT&Tag (Yeasen Biotechnology, 12597ES48) following the manufacturer’s protocol. Briefly, 1 × 10^5^ target cells were mixed with 10% Drosophila S2 cells and incubated with 10 µL of Concanavalin A beads. Lung cancer cells (shControl and shKMT2D) were incubated overnight at 4 °C with 1 µg of primary antibody (H3K4me1, H3K27ac, H3K27me3, KMT2D, p300, EZH2) and internally control Drosophila-specific histone variant H2Av antibody, followed by a 1 h incubation with 1:100 diluted secondary antibody at room temperature. The pA-Tn5 transposase adapter complex was then added and incubated for 1 h at room temperature. DNA was purified using Hieff NGSTM DNA Selection Beads and amplified with 5 initial PCR cycles, with additional cycles determined by qPCR. Libraries were sequenced on the NovaSeq platform. Sequencing reads were trimmed, quality-controlled, and aligned to the human genome (GRCh37/hg19), as previously described [31].

### Statistical analysis

All statistical analyses were performed using standard methods and independently repeated at least three times unless otherwise specified. Data are presented as mean ± standard deviation (SD) as indicated in the figure legends. Two-group comparisons used unpaired two-tailed Student’s t-tests (Welch’s t-test if variances were unequal). While one-way or two-way Analysis of Variance (ANOVA) with a post hoc test was used for analyses among three or more groups, as indicated. Correlations were assessed using Pearson’s correlation coefficient (*r*) or Spearman’s rank correlation (*ρ*), selected based on data distribution and linearity. *P* < 0.05 was considered statistically significant. Statistical tests used for each figure are detailed in the corresponding legends.

## Supplementary information

CDD-25-3285RR_Supplementary Figures and Legends

Supplementary Table S1

Supplementary Table S2

Supplementary Table S3

Supplementary Table S4

Supplementary Table S5

Supplementary Table S6

CDD-25-3285RR_Original Data File 1

## Data Availability

RNA-seq, ATAC-seq, and CUT&Tag sequencing data generated in this study have been deposited in the Genome Sequence Archive (GSA) [93] at the National Genomics Data Center [94], China National Center for Bioinformation/Beijing Institute of Genomics, Chinese Academy of Sciences, under accession numbers HRA013915, HRA013916, HRA013918, and HRA013993. This study does not report original code. Any additional information required to reanalyze the data reported in this paper is available from the lead contact upon request.

## Materials availability

All plasmids and stable cell lines relevant to this manuscript are available from the lead contact with a completed materials transfer agreement.
