# Supplementary material for: KMT2D loss drives adeno-to-squamous transition and sensitizes TKI-resistant lung cancer to AURKA inhibition
Source: Cell Death Differ. 2026 Jan 8;33(7):1416–35. doi: 10.1038/s41418-025-01657-7 (PMC13342116; doi:10.1038/s41418-025-01657-7)
Supplement: Supplementary file 1 — CDD-25-3285RR_Supplementary Figures and Legends [file 41418_2025_1657_MOESM1_ESM.pdf]

Supplementary Materials for

KMT2D Loss Drives Adeno-to-Squamous Transition and Sensitizes TKI-Resistant Lung Cancer to AURKA Inhibition

Running title:

KMT2D Deficiency Links Squamous Transition to Mitotic Kinase Vulnerability

Nana Chen[1,2,11], Mouxiang Fang[1,3,11], Leqi Zhong[1,11], Xiaolong Li[4], Yijia Zhou[5], Jianhua Zhan[1], Manli Wang[6], Zhaoyuan Fang[7], Hua Wang[5], Shijie Tang[8], Fang Liu[1], Bing Deng[1], Ning Chen[1], Jie Lei[1], Yuchen Zhang[1], Min Yan[1], Zhengzhi Zou[3], Yijun Gao[1], Chong Chen[6], Wenzhao Zhong[2], Srinivas Vinod Saladi[9], Hongbin Ji[5,10,12*], Quentin Liu[1,4,12*], Zifeng Wang[1,12*], Bin He[1,12*]

1 State Key Laboratory of Oncology in South China, Guangdong Provincial Clinical Research Center for Cancer, Psychobehavioral Cancer Research Center, Sun Yat-sen University Cancer Center, Guangzhou 510060, China.

2 Guangdong Lung Cancer Institute, Guangdong Provincial Key Laboratory of Translational Medicine in Lung Cancer, Guangdong Provincial People's Hospital, Guangdong Academy of Medical Sciences, Southern Medical University, Guangzhou 510080, China.

3 MOE Key Laboratory of Laser Life Science & Guangdong Provincial Key Laboratory of Laser Life Science, College of Biophotonics, South China Normal University, Guangzhou 510631, China.

4 Institute of Cancer Stem Cell, Dalian Medical University, Dalian 116044, China.

5 State Key Laboratory of Cell Biology, Shanghai Institute of Biochemistry and Cell Biology, Center for Excellence in Molecular Cell Science, Chinese Academy of Sciences, Shanghai 200031, China.

6 State Key Laboratory of Biotherapy and Cancer Center, West China Hospital, Sichuan University, Chengdu 610041, China.

7 Department of Colorectal Surgery and Oncology, the Second Affiliated Hospital, and Center for Biomedical Systems and Informatics, Zhejiang University-University of Edinburgh Institute (ZJU-UoE Institute), Zhejiang University School of Medicine, Zhejiang University, Hangzhou 310000, Zhejiang, China.

8 Shanghai General Hospital, Shanghai Jiao Tong University School of Medicine, Shanghai 200025, China.

9 Department of Cell and Cancer Biology, University of Toledo, College of Medicine and Life Sciences, Toledo, OH 43614, USA.

10 School of Medicine, Westlake University, Hangzhou 310024, China.

11 These authors contributed equally

12 Corresponding authors

Email: jihongbin@westlake.edu.cn; liuq9@mail.sysu.edu.cn; wzifeng@mail.sysu.edu.cn; hebin@sysucc.org.cn

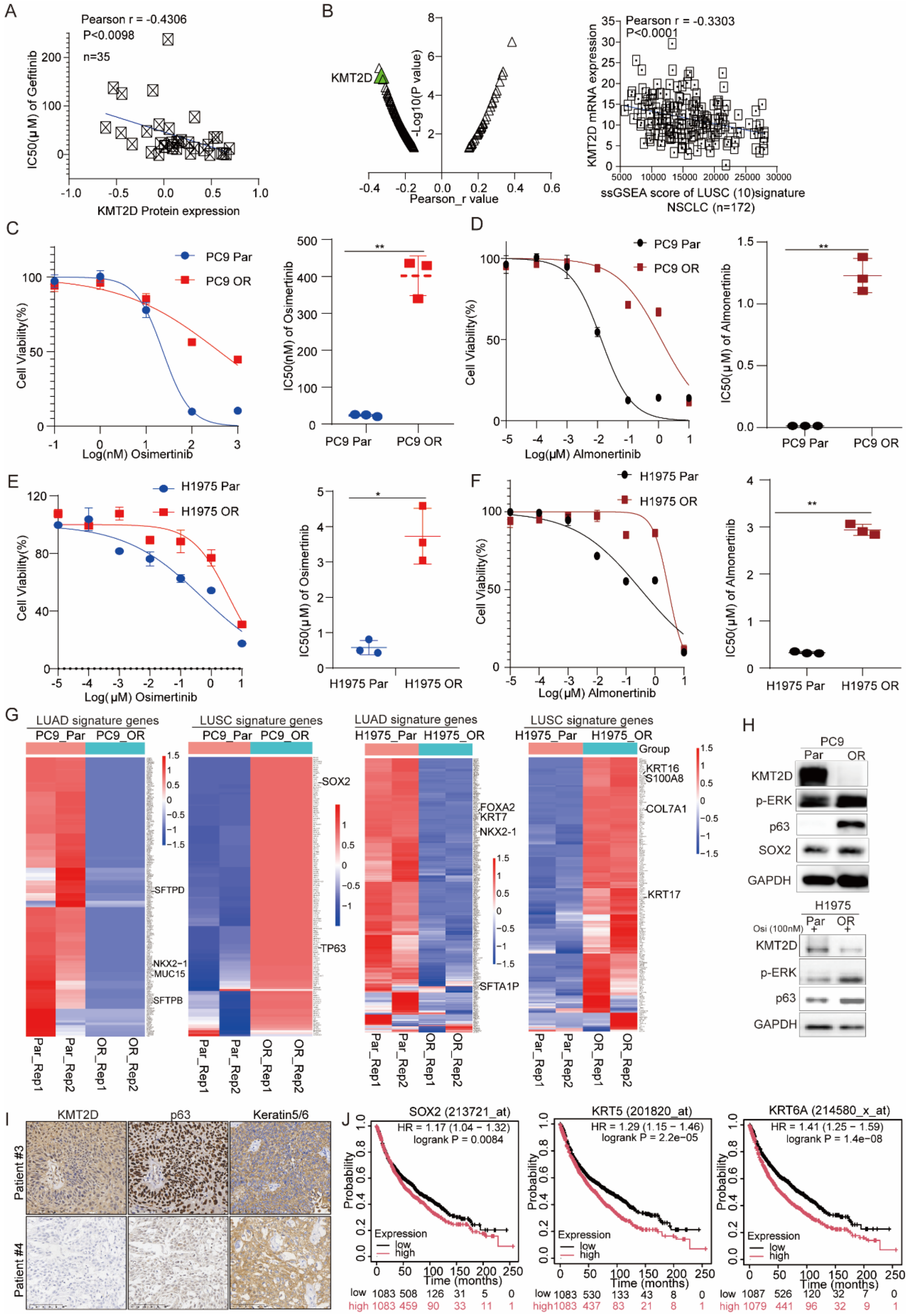

Supplementary Fig.1 KMT2D low expression is associated with tyrosine kinase inhibitor (TKI) resistance and squamous phenotype transition.

(A) Pearson correlation analysis between KMT2D protein expression and gefitinib IC_{50} values across 35 NSCLC cell lines (r =-0.4306, *P* < 0.0098).

(B) Volcano plot of Pearson correlation coefficients between expression of 349 epigenetic

regulators (filtered for $p < 0.05$) and ssGSEA-derived squamous signature scores (10-gene LUSC signature) in CCLE NSCLC cell lines (n = 172). KMT2D is highlighted in green. Right: Correlation between KMT2D mRNA expression and the ssGSEA squamous signature score (10-gene LUSC signature) in CCLE NSCLC lines (r = -0.3303, $P < 0.0001$).

(C-D) Dose-response curve showing the percentage of cell viability or statistical analysis of IC_{50} values in PC9 Parental (Par) cells and osimertinib-resistant (OR) PC9 cells after treatment with increasing concentrations of osimertinib or almonertinib. IC_{50} comparisons were performed on $\log_{10}(IC_{50})$ using two-tailed Welch's t-test, n=3, $^{**}P < 0.01$.

(E-F) Dose-response curve showing the percentage of cell viability or statistical analysis of IC_{50} values in H1975 Parental (Par) cells and Osimertinib-resistant (OR) H1975 cells after treatment with increasing concentrations of osimertinib or almonertinib. Two-tailed Welch's t-test, n=3, $^{*}P < 0.05$, $^{**}P < 0.01$.

(G) Heatmaps displaying the expression of LUAD (lung adenocarcinoma) signature genes (left) and LUSC (lung squamous cell carcinoma) signature genes (right) in PC9/H1975_Par (Parental) and PC9/H1975_OR (osimertinib-resistant) cells. Gene expression values are Z-score normalized, with red indicating high expression and blue indicating low expression.

(H) Western blot analysis of KMT2D, phosphorylated ERK (pERK), and p63 (ΔNp63) in parental (Par) and osimertinib-resistant (OR) PC9 and H1975 cells. GAPDH serves as a loading control.

(I) Representative IHC in clinical specimens. Two NSCLC cases stained for KMT2D, p63 (ΔNp63), and Keratin 5/6. Scale bars, 100 μm.

(J) Kaplan-Meier survival curves of lung cancer patients stratified by SOX2, KRT5, and KRT6A expression levels.

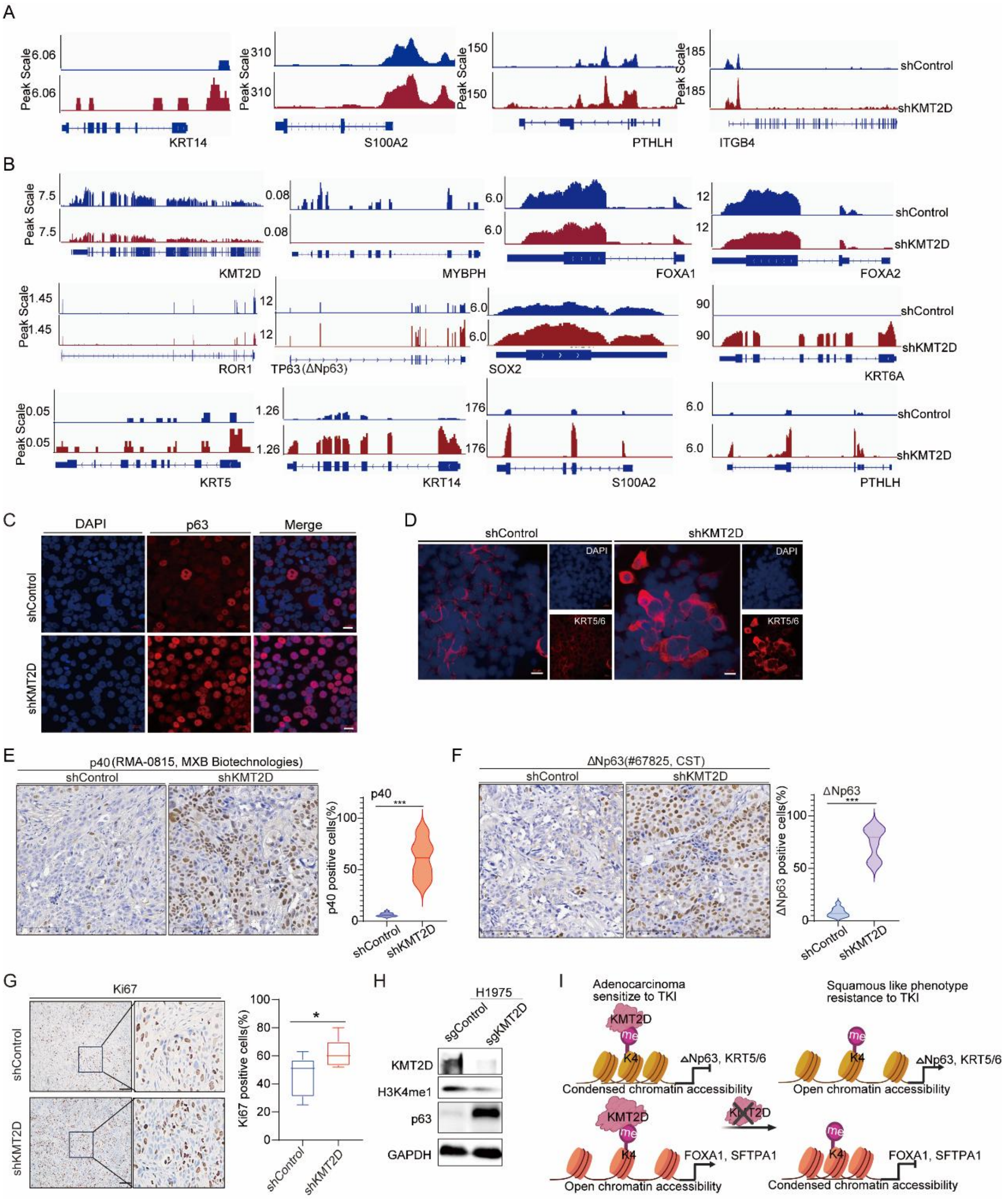

Supplementary Fig.2 KMT2D deficiency drives the transition from lung adenocarcinoma to a squamous-like phenotype through epigenetic chromatin rewiring.

(A) ATAC-seq IGV plots of key genes involved in squamous differentiation following KMT2D knockdown. Peaks represent binding regions in shControl (blue) and shKMT2D (red) conditions.

(B) IGV plots of RNA-seq data showing the expression of adenocarcinoma- and squamous lineage-related genes in shControl (blue) and shKMT2D (red) groups.

(C-D) Representative immunofluorescence staining of the p63 and KRT5/6 in shControl and shKMT2D lung cells. Blue: DAPI (nuclei); Red: p63 or KRT5/6. Scale bars, 20μm.

(E-F) Representative immunohistochemistry (IHC) images and quantification of p40 (E) and ΔNp63 (F) expression in xenograft tumors derived from H358 cells expressing

shControl or shKMT2D. Quantification of positive cells (%) per tumor (n = 5). Data are presented as mean±SD. Two-tailed t-test. ***P < 0.001. Scale bars, 100μm.

(G) Immunohistochemical (IHC) staining for Ki67 in xenograft tumors bearing shControl or shKMT2D cells. Boxed regions are shown at higher magnification to highlight Ki67-positive nuclei. Quantification of Ki67-positive cells (percentage of nuclei) is summarized in the box plot at right (n = 5). Data are presented as mean±SD. Two-tailed t-test, *P< 0.05. Scale bars: 100μm.

(H) Representative western blot of KMT2D, H3K4me1, and p63 (ΔNp63) in EGFR-mutant H1975 cells after KMT2D knockout. Cells were transduced with non-targeting control (sgControl) or KMT2D-targeting sgRNA (sgKMT2D), GAPDH served as the loading control.

(I) Schematic model illustrating KMT2D in modulating chromatin states with adenocarcinoma and squamous phenotypes in NSCLC (Created with BioRender.com).

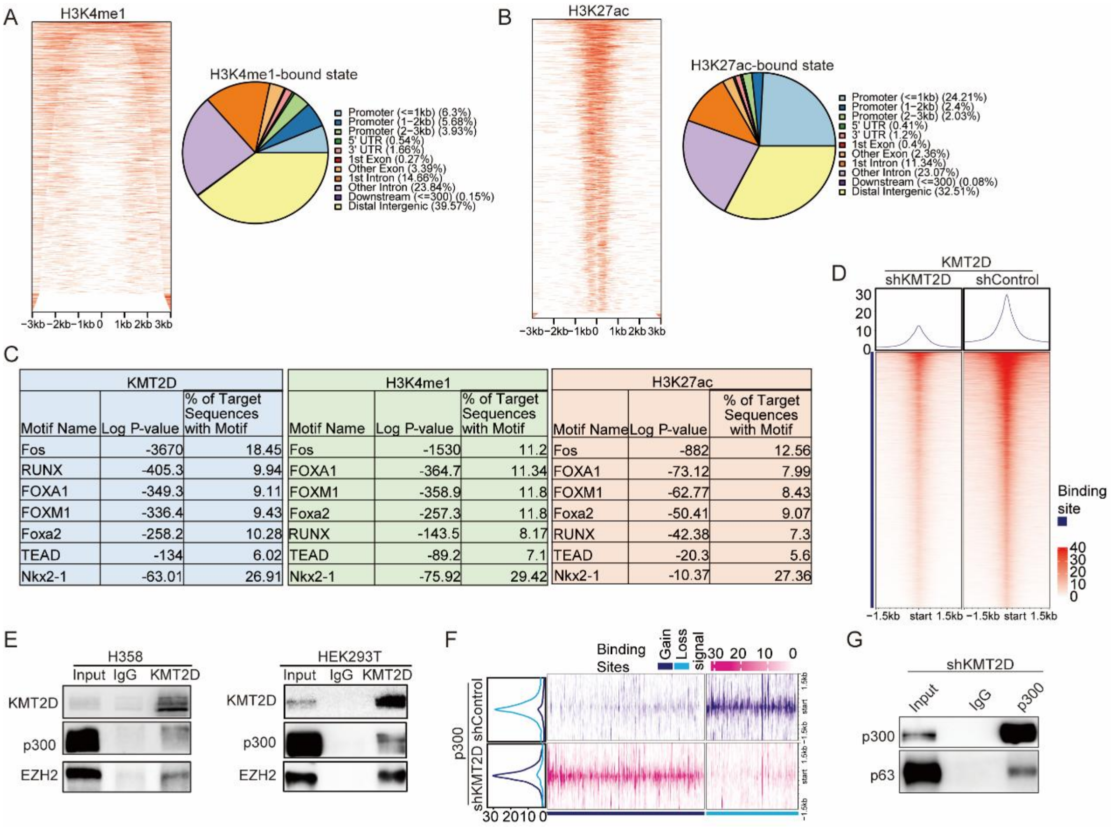

KMT2D			H3K4me1			H3K27ac		
Motif Name	Log P-value	% of Target Sequences with Motif	Motif Name	Log P-value	% of Target Sequences with Motif	Motif Name	Log P-value	% of Target Sequences with Motif
Fos	-3670	18.45	Fos	-1530	11.2	Fos	-882	12.56
RUNX	-405.3	9.94	FOXA1	-364.7	11.34	FOXA1	-73.12	7.99
FOXA1	-349.3	9.11	FOXM1	-358.9	11.8	FOXM1	-62.77	8.43
FOXM1	-336.4	9.43	Foxa2	-257.3	11.8	Foxa2	-50.41	9.07
Foxa2	-258.2	10.28	RUNX	-143.5	8.17	RUNX	-42.38	7.3
TEAD	-134	6.02	TEAD	-89.2	7.1	TEAD	-20.3	5.6
Nkx2-1	-63.01	26.91	Nkx2-1	-75.92	29.42	Nkx2-1	-10.37	27.36

Supplementary Fig.3 Loss of KMT2D interrupts the epigenetic crosstalk required to maintain lineage fidelity.

(A-B) Heatmaps of H3K4me1 and H3K27ac signal around transcription start sites (±3 kb) in KMT2D-WT lung cancer cells. Pie charts show genomic distribution of H3K4me1 and H3K27ac peaks across promoters, exons, introns, and distal intergenic regions.

(C) Enrichment of binding motifs at KMT2D, H3K4me1, and H3K27ac binding sites in WT cells.

(D) Heatmaps depicting KMT2D signal intensity in shKMT2D and shControl cells across ±1.5 kb binding sites.

(E) Co-immunoprecipitation (co-IP) assays in H358 and HEK293T cells. KMT2D was immunoprecipitated from whole cell lysates, and immunoblotting with p300 and EZH2. IgG was used as a negative control, and input lysates served as loading controls.

(F) Heatmaps depicting p300 signal intensity in shControl and shKMT2D cells across ±1.5 kb binding sites.

(G) Co-immunoprecipitation analysis of p300-associated protein complexes in H358 shKMT2D cells. Cell lysates were immunoprecipitated using anti-p300 antibody or control IgG, followed by immunoblotting with antibodies against p63 (ΔNp63) and p300.

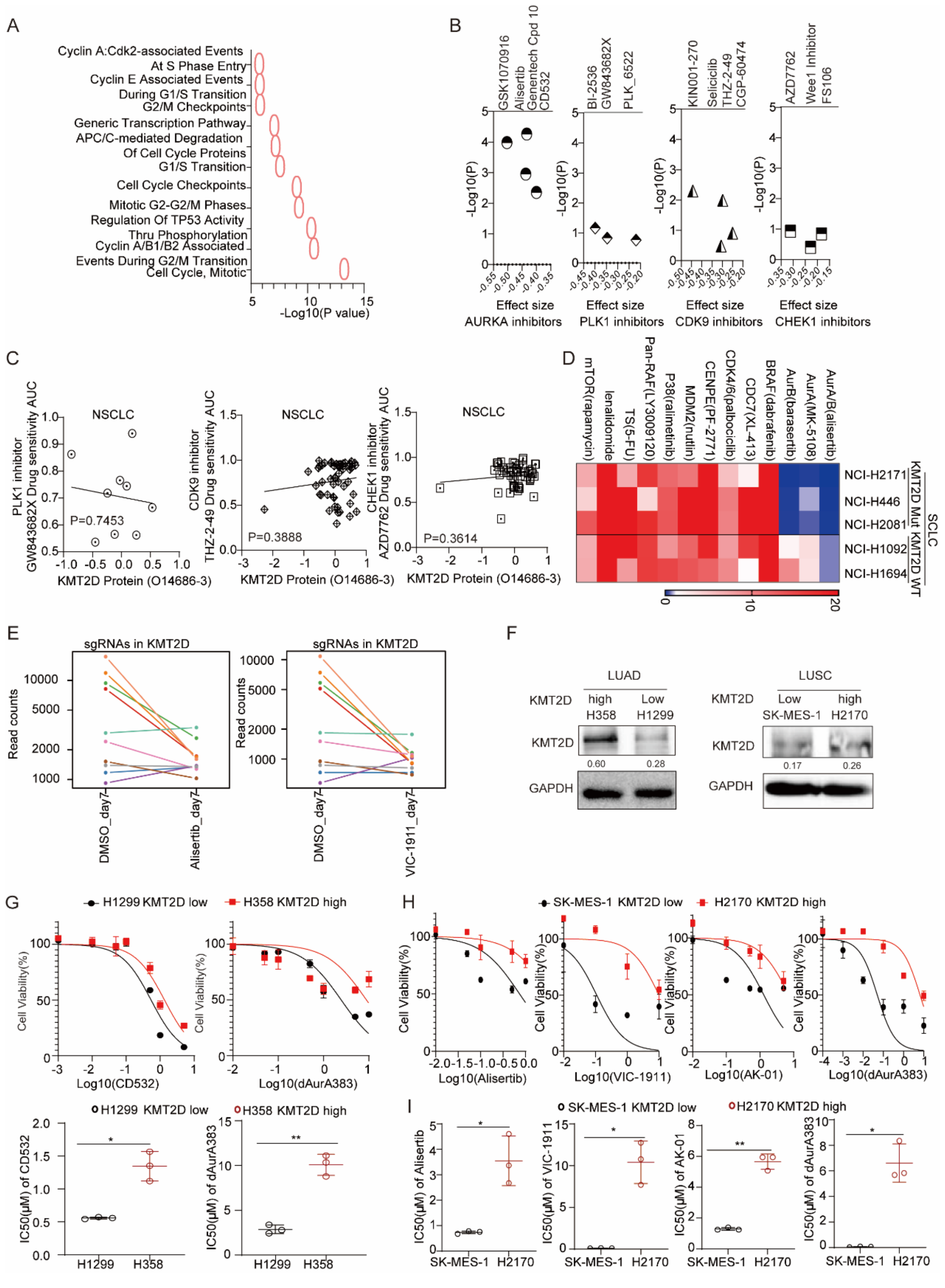

Supplementary Fig.4 Kinome-wide CRISPR-Cas9 knockout screening revealed AURKA as a therapeutic target for KMT2D deficient lung cancer.

(A) Pathway enrichment of the essential genes using enrichr analysis.

(B) Drug sensitivity profiles in KMT2D Mutant pan-cancer cells. Scatter plots display the -log(P) values (y-axis) against effect sizes (x-axis) for AURKA, PLK1, CDK9, and CHEK1 inhibitors.

(C) Scatter plots showing the relationship between KMT2D protein levels (O14686-3) and

drug sensitivity (AUC) to inhibitors of PLK1 (GW843682X, left), CDK9 (THZ-2-49, middle), and CHEK1 (AZD7762, right) across NSCLC cell lines.

(D) Heatmap depicting the differential sensitivity of small cell lung cancer (SCLC) cell lines to various drugs, stratified by KMT2D mutation status. Drug sensitivity is represented as a color gradient, where red indicates low sensitivity (higher IC_{50} or lower drug efficacy) and blue indicates higher sensitivity (lower IC_{50} or high drug efficacy).

(E) Read count analysis for CRISPR sgRNAs targeting KMT2D in NSCLC cells treated with DMSO or AURKA inhibitor.

(F) Western blot analysis showing KMT2D protein expression levels in representative lung adenocarcinoma (LUAD) and lung squamous cell carcinoma (LUSC) cell lines. Relative KMT2D protein intensities (normalized to GAPDH) are indicated below each band.

(G) Dose-response curves illustrating the effect of AURKA allosteric inhibitor (CD532) and AURKA degrader (dAurk383) on cell viability in KMT2D-high and -low lung adenocarcinoma cell lines. Statistical analysis of IC_{50} values for CD532 and dAurA383 was indicated (n=3). *P<0.05; **P<0.01.

(H) Dose-response curves illustrating the effect of AURKA inhibitors (alisertib, VIC-1911, AK-01), and AURKA degrader(dAurk383) on cell viability in KMT2D-high and -low lung squamous cell lines.

(I) Statistical analysis of IC_{50} values for alisertib, VIC-1911, AK-01, and dAurA383 in KMT2D-high and KMT2D-low lung squamous cells (n = 3 independent biological replicates per condition). Data are presented as mean ± SD; two-tailed t-test, *P<0.05; **P<0.01.

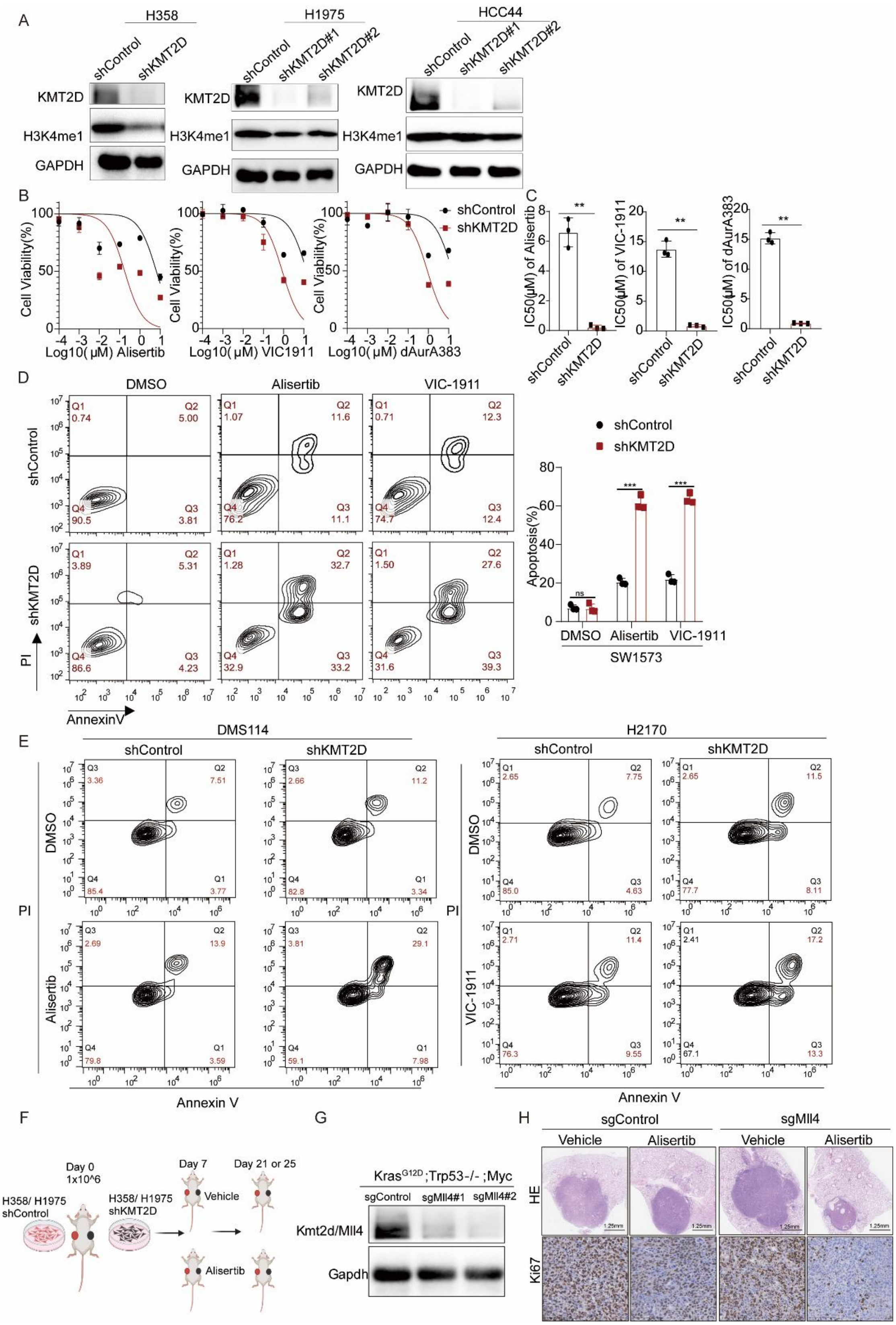

Supplementary Fig.5 KMT2D loss confers vulnerability to AURKA inhibition both *in vitro* and *in vivo*.

(A) Western blot analysis of KMT2D, H3K4me1, and GAPDH (as a loading control) across lung cancer cell lines: H358, H1975, and HCC44. Cells were infected with either

shControl or shKMT2D.

(B) Dose-response curves of cell viability in NSCLC(H358) cells treated with AURKA inhibitors (alisertib, VIC-1911) and the AURKA degrader dAurA383. Cells were infected with either control shRNA (shControl, black) or KMT2D-targeting shRNA (shKMT2D, red). Cell viability was assessed after treatment with increasing concentrations of the drugs.

(C) IC_{50} values for alisertib, VIC-1911, and dAurA383 in shControl versus shKMT2D cells. Data are presented as mean±SD, n=3, two-tailed t-test, **P < 0.01.

(D-E) Representative flow cytometry analysis of Annexin V/PI staining in SW1573 (LUAD), DMS114 (SCLC), and H2170 (LUSC) cells transduced with shControl or shKMT2D and treated with DMSO or AURKA inhibitors (alisertib or VIC-1911). Dot plots show Annexin V vs PI with quadrant gating. Statistical comparisons were performed using two-way ANOVA. ***P < 0.001; ns, not significant.

(F) Schematic overview of xenograft model (Created with BioRender.com).

(G) Western blot analysis showing the expression levels of Kmt2d/Mll4 in $Kras^{G12D}$; $Trp53^{-/-}$; *Myc* organoids. Organoids were infected with Control sgRNA (sgCon) or two different sgRNAs targeting Kmt2d/Mll4 (sgMll4#1 and sgMll4#2). Gapdh was used as a loading control to confirm equal protein loading across samples.

(H) The representative H&E staining (Scale bar, 1.25mm) and Ki67 (Scale bar, 100μm) IHC staining of the lung cancer in veh-treated and alisertib-treated tumors.

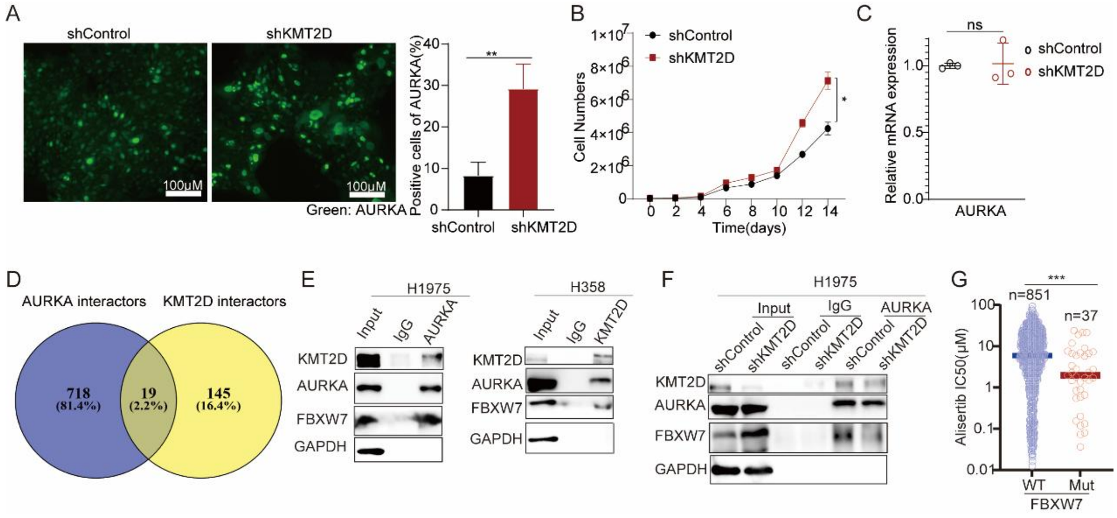

Supplementary Fig.6 KMT2D loss stabilizes AURKA by inhibiting FBXW7-mediated degradation.

(A) Left: Representative immunofluorescence images showing the expression of AURKA (green) in shControl and shKMT2D lung cancer cells. Scale bars, 100μm. Right: Percentage of AURKA-positive cells (mean ± SD, n = 5), two-tailed t-test, ***P* < 0.01.

(B) Comparison of shControl and shKMT2D growth curves in H358 cells. Cells were harvested and counted at different time points (mean ± SD, n=3). Statistical comparison at day 14 was performed using an unpaired two-tailed t-test (shKMT2D vs. shControl), **P*<0.05

(C) qRT-PCR analysis of AURKA transcript levels in shControl or shKMT2D cells. Relative AURKA mRNA expression was normalized to 18s and presented as mean±SD from three independent experiments. Two-tailed t-test, ns, no significant.

(D) Venn diagram showing the overlap between reported AURKA interactors and KMT2D interactors curated from the BioGRID database.

(E) Representative co-immunoprecipitation (Co-IP) assays were performed in H1975 (left) and H358 (right) cells. Immunoprecipitation with anti-AURKA antibody (H1975) or anti-KMT2D antibody (H358) followed by immunoblotting with KMT2D, AURKA, and FBXW7. IgG was used as a negative control, and GAPDH served as a loading control.

(F) Representative Co-IP was performed in H1975 shControl and shKMT2D cells using anti-AURKA antibody or IgG control, followed by immunoblotting with KMT2D, AURKA, and FBXW7. Input lysates and IgG pulldowns served as controls. GAPDH was used as a loading control.

(G) Genomics of Drug Sensitivity in Cancer (GDSC) dose response data comparing alisertib sensitivity in FBXW7 wild-type (WT) versus mutant (Mut) cell lines. Each point is the IC_{50} (μM) for one cell line. Two-tailed t-test, ****P*<0.001.

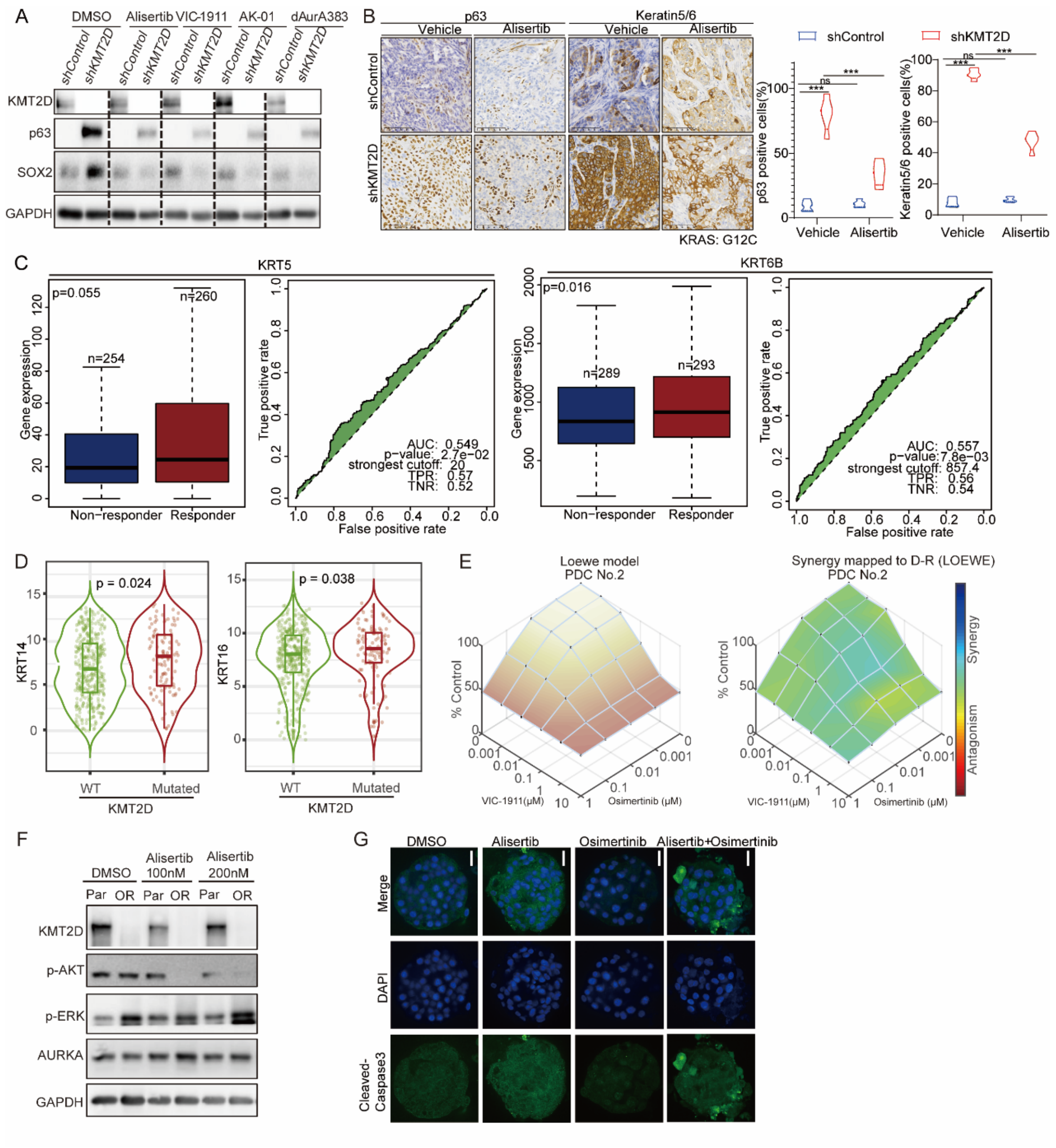

Supplementary Fig.7 AURKA inhibition impairs squamous identity and overcomes TKI resistance in NSCLC.

(A) Western blot analysis of squamous lineage-related protein expression in H1975 lung cancer cells, with and without KMT2D knockdown, following 72-hour treatment with AURKA inhibitors.

(B) Immunohistochemical (IHC) analysis of p63 (left panels) and keratin 5/6 (right panels) expression in xenograft tumor sections derived from either control shRNA (shControl) or shRNA targeting KMT2D (shKMT2D) NSCLC cells with KRAS(G12C) mutation, treated with vehicle or the AURKA inhibitor alisertib. Scale bar: 100μm. The box plots (right) quantify the percentage of p63 or KRT5/6 positive cells under each condition, 5-10 random HPFs averaged, two-way ANOVA, ****P*<0.001; ns, not significant (n = 4 or 5 tumors/group).

(C) Boxplot comparing the expression of KRT5 and KRT6B (lung squamous associated genes marker) between non-responders (blue) and responders (red) to the AURKA

inhibitor Alisertib. Receiver operating characteristic (ROC) curve evaluating the predictive power of KRT5 and KRT6B expression in distinguishing responders from non-responders. Data was obtained from solid tumor cells and analyzed with rocplot.com.

(D) Analysis the expression of squamous lineage markers KRT14 and KRT16 in KMT2D wild-type (WT) versus KMT2D-mutated samples in non-small cell lung cancer (NSCLC). The data obtained from TCGA and analyzed by TIMER.

(E) Synergy analysis of the combination effect of osimertinib and VIC-1911 in TKI resistant patient derived cells (PDC. No.2).

(F) Western blot analysis of KMT2D, phosphorylated AKT (p-AKT), phosphorylated ERK (p-ERK), and AURKA in parental (Par) and osimertinib resistant (OR) cells following treatment with DMSO (control), 100 nM alisertib, or 200 nM alisertib. GAPDH serves as a loading control.

(G) Representative immunofluorescence images of NSCLC organoid (PDC. No.2) treated with DMSO (control), alisertib (AURKA inhibitor), osimertinib (TKi), or the combination of alisertib and osimertinib. Organoids were stained for Cleaved-Caspase 3 (green) and DAPI (blue). Scale bar, 20μm.
