# Supplementary material for: KMT2D loss drives adeno-to-squamous transition and sensitizes TKI-resistant lung cancer to AURKA inhibition
Source: Cell Death Differ. 2026 Jan 8;33(7):1416–35. doi: 10.1038/s41418-025-01657-7 (PMC13342116; doi:10.1038/s41418-025-01657-7)

Supplementary Fig.1H

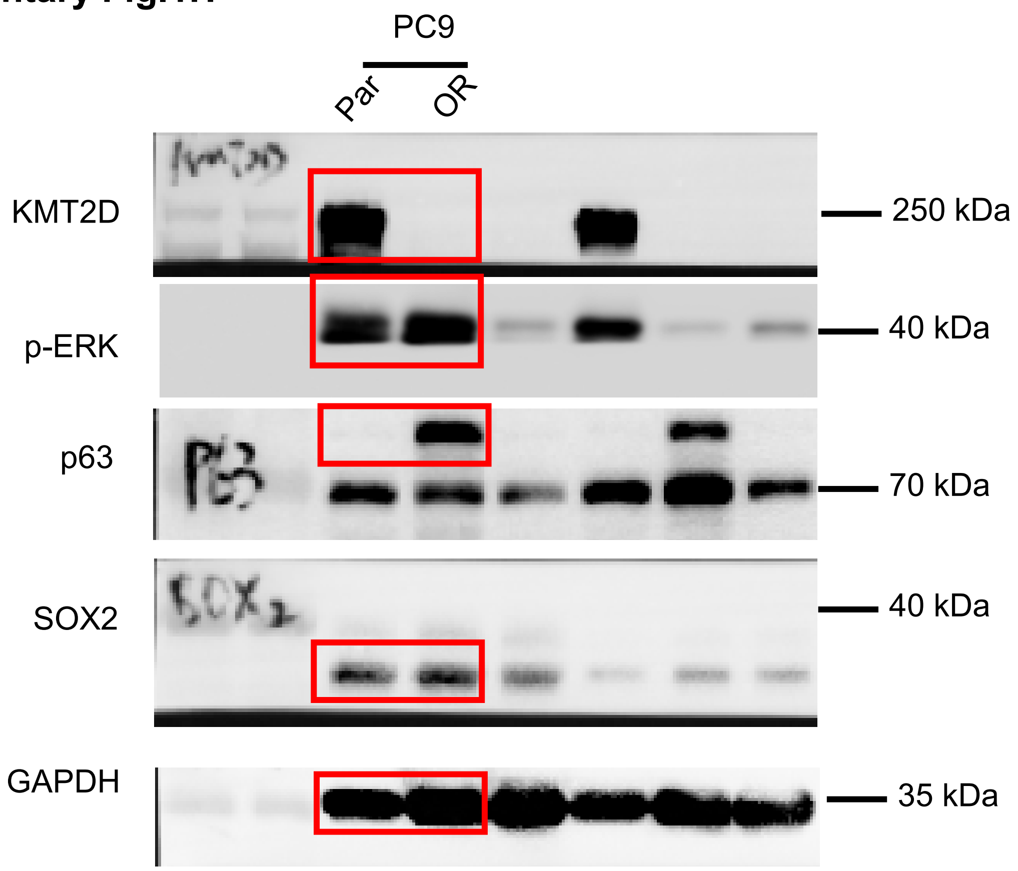

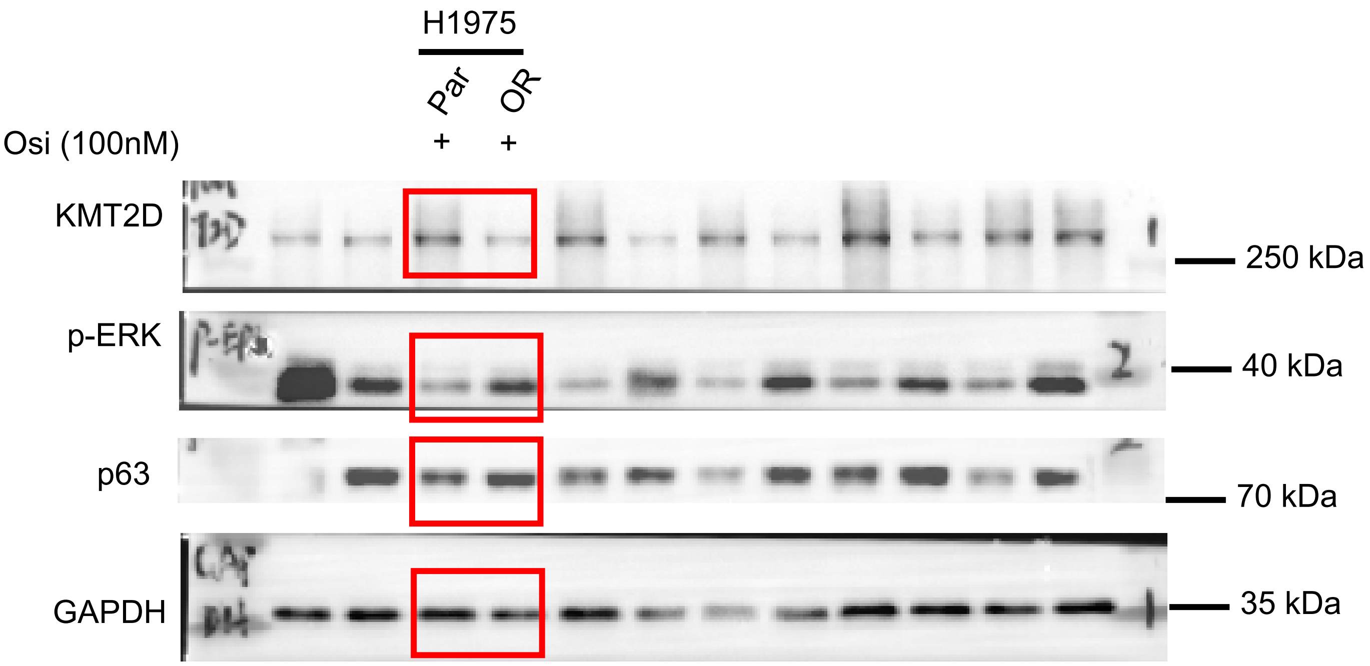

Supplementary Fig.2H

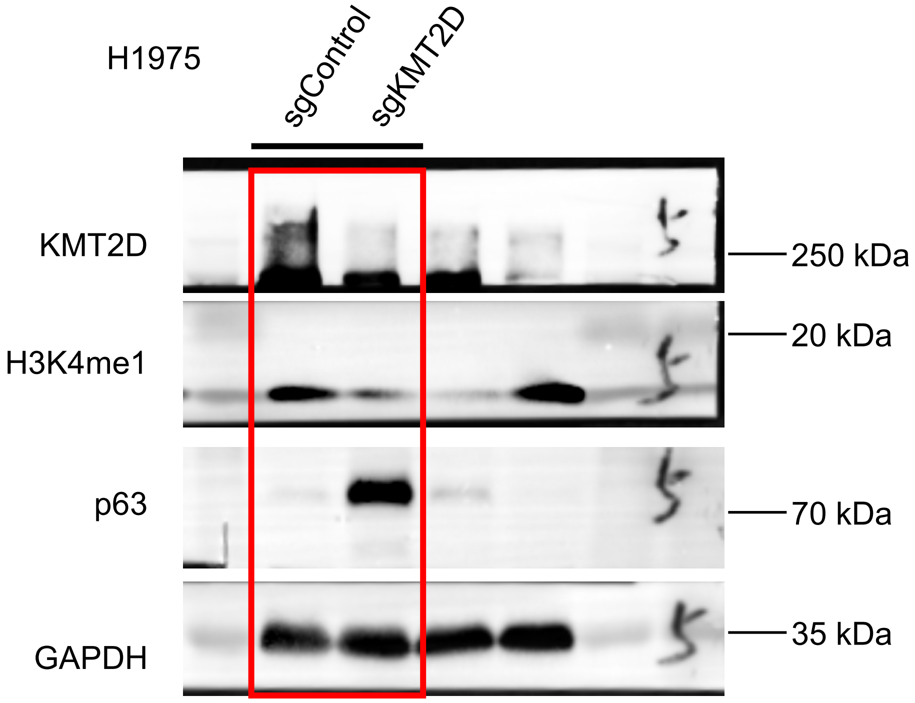

Fig.3J

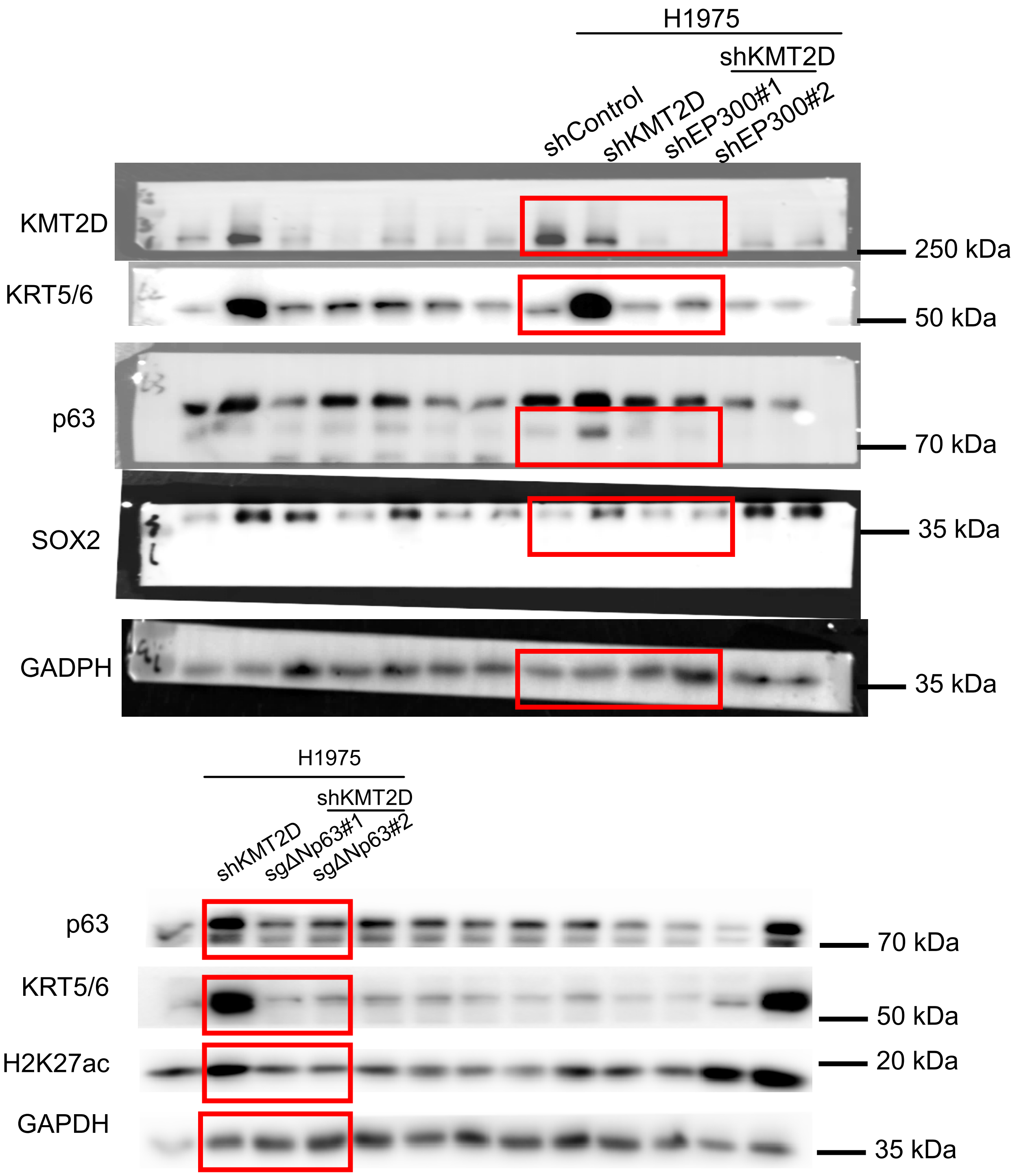

Supplementary Fig.3E

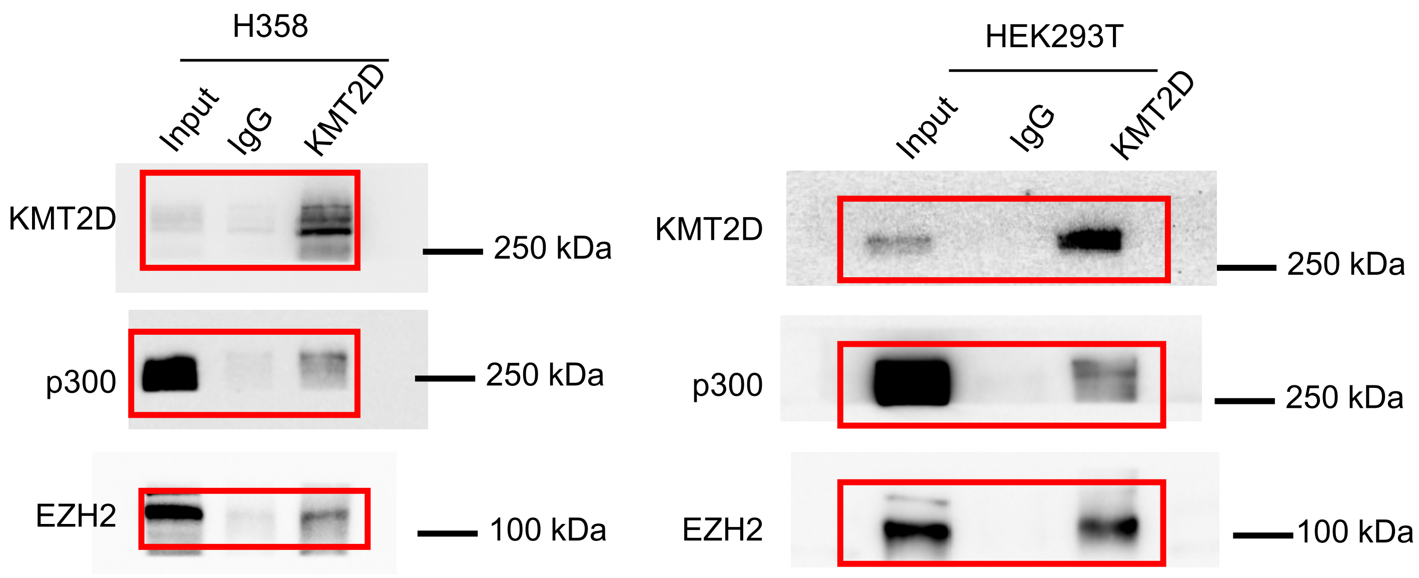

Supplementary Fig.3G

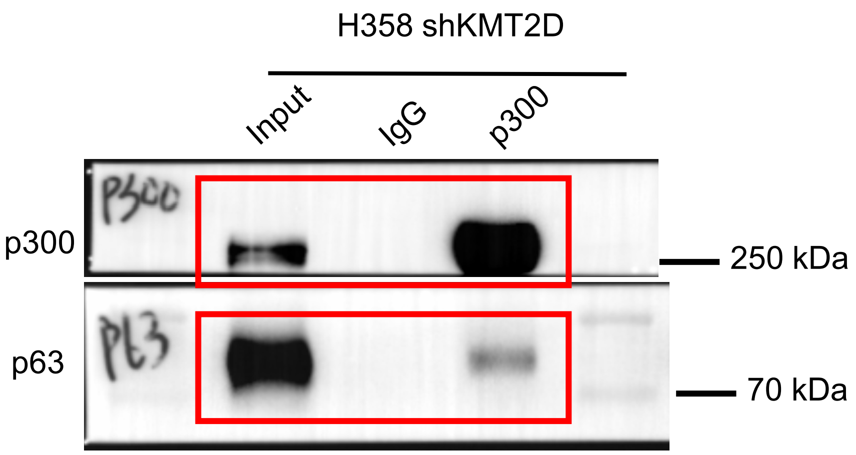

Supplementary Fig.4F

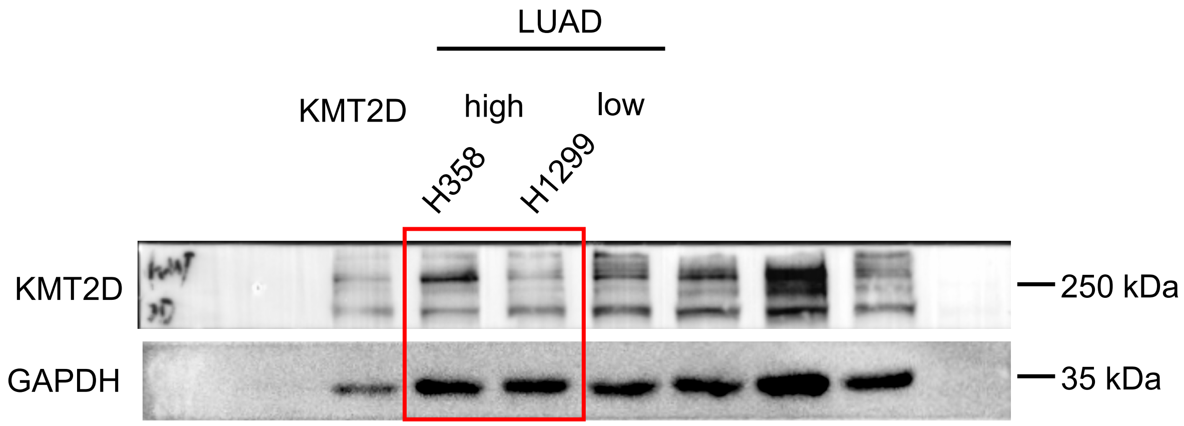

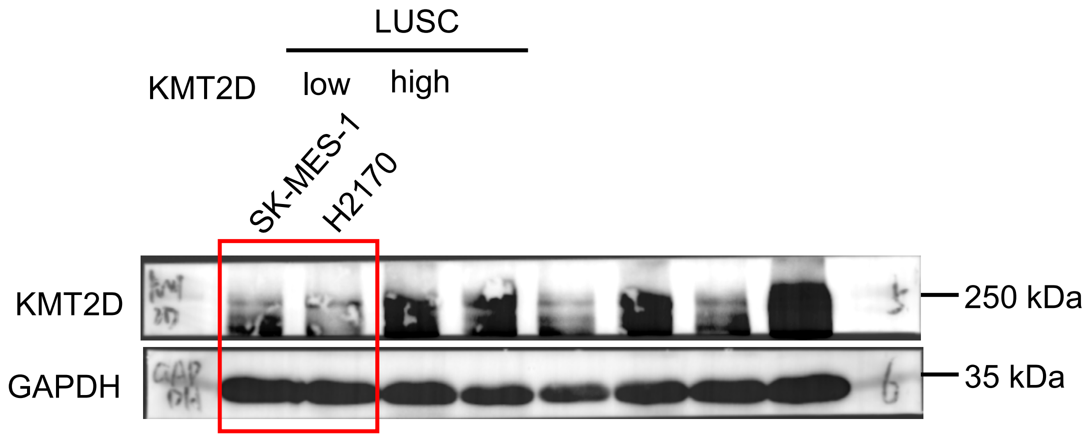

Supplementary Fig.5A

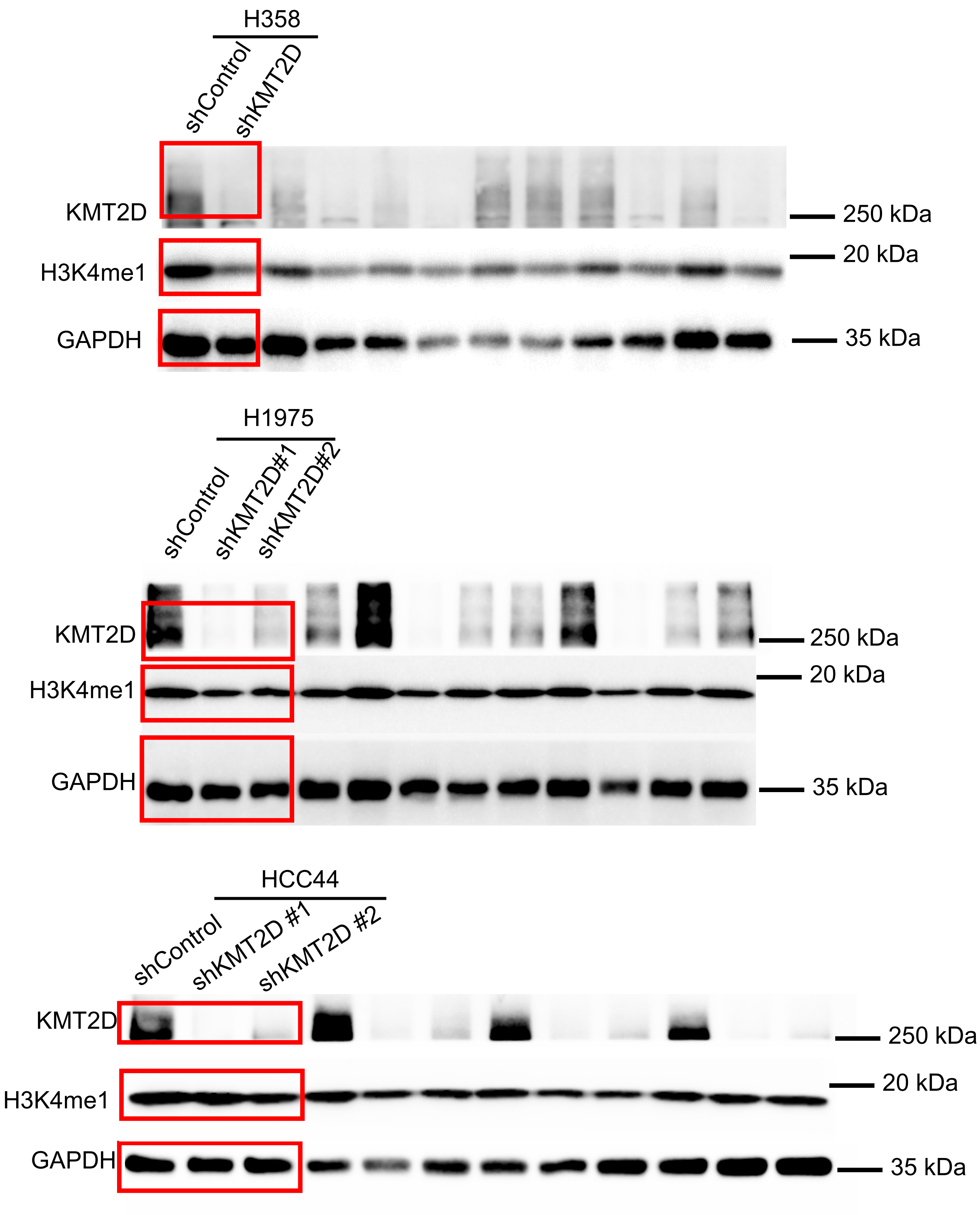

Supplementary Fig.5G

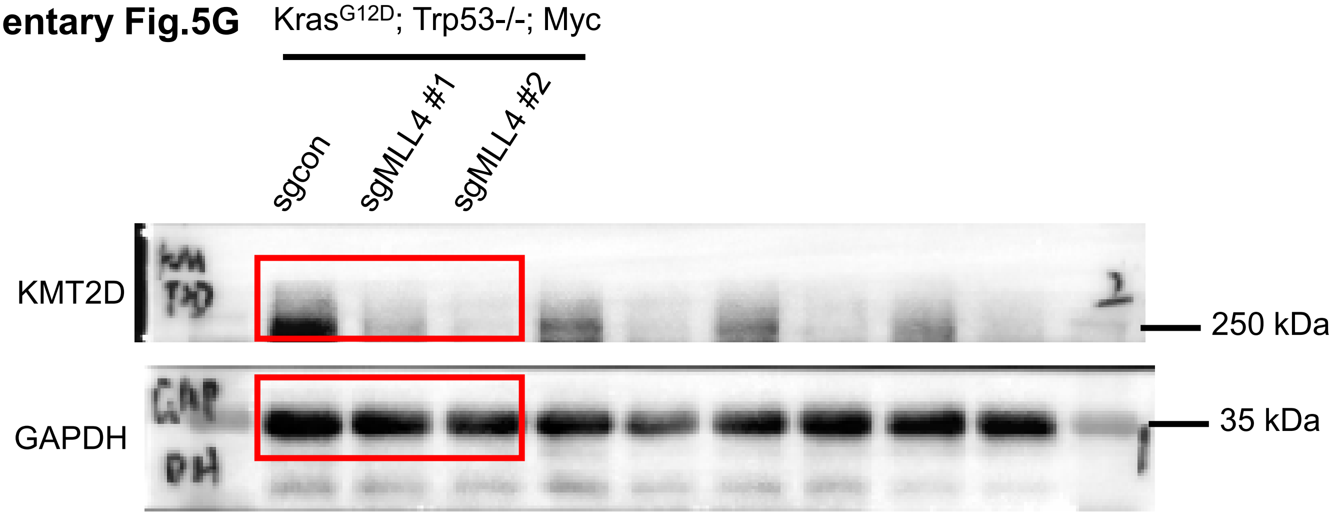

Fig.6A

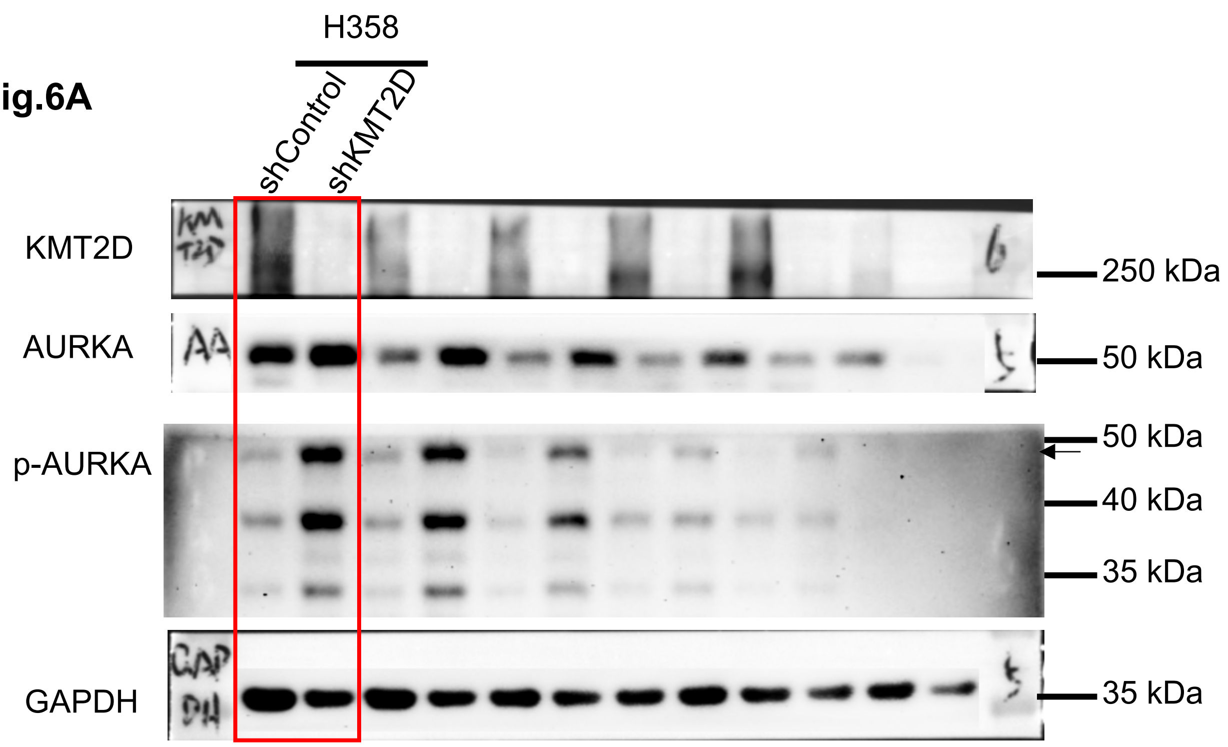
H358
shControl
shKMT2D
KMT2D
AURKA
p-AURKA
GAPDH
250 kDa
50 kDa
50 kDa
40 kDa
35 kDa
35 kDa

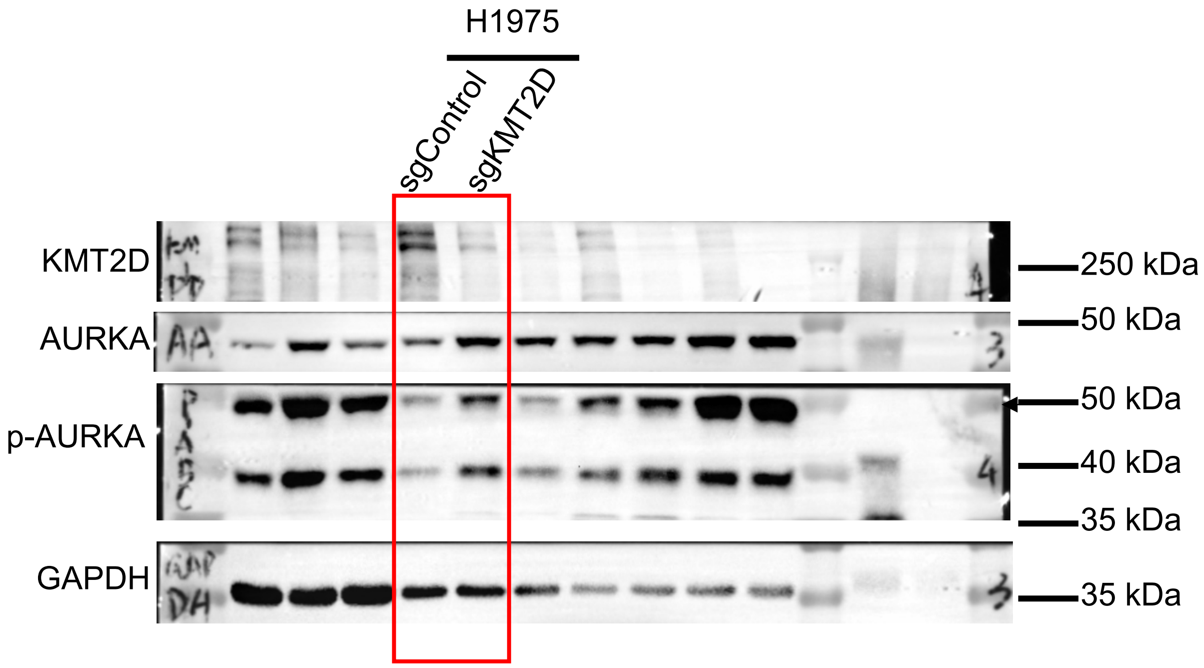
H1975
sgControl
sgKMT2D
KMT2D
AURKA
p-AURKA
GAPDH
250 kDa
50 kDa
50 kDa
40 kDa
35 kDa
35 kDa

Fig.6B

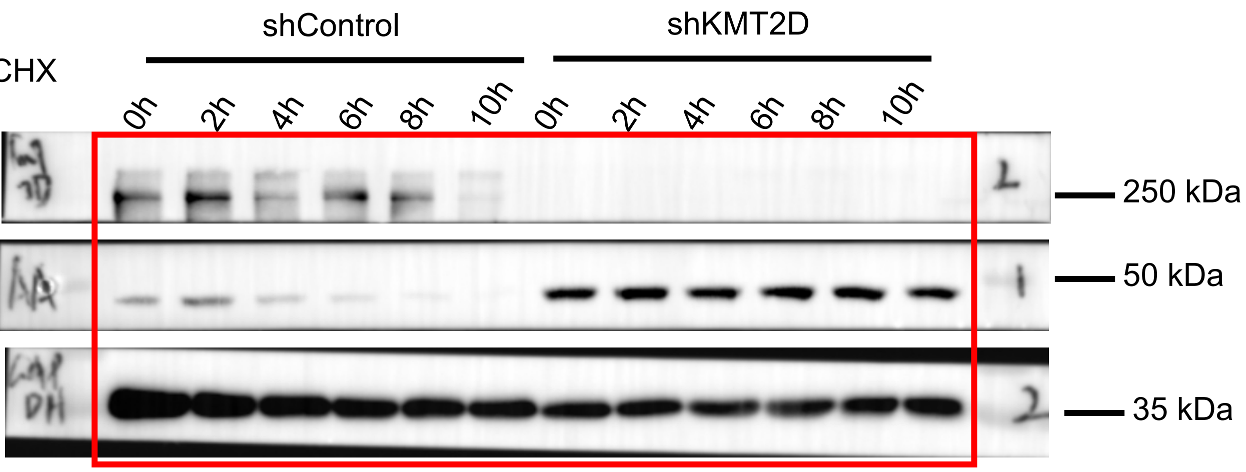

Fig.6C

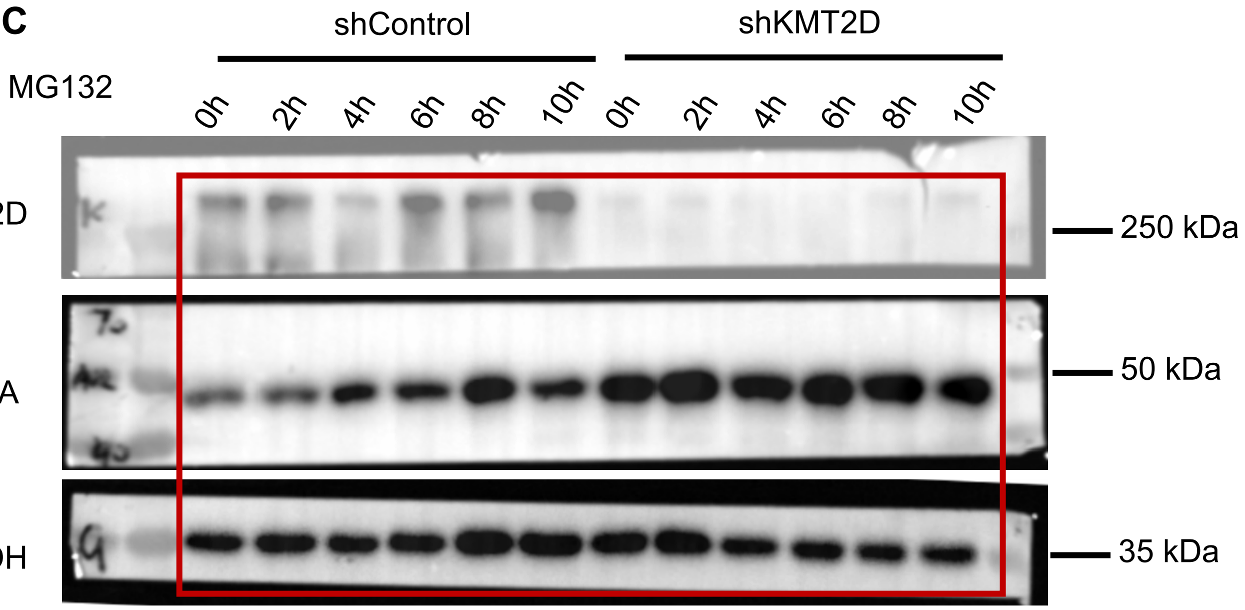

Fig.6D

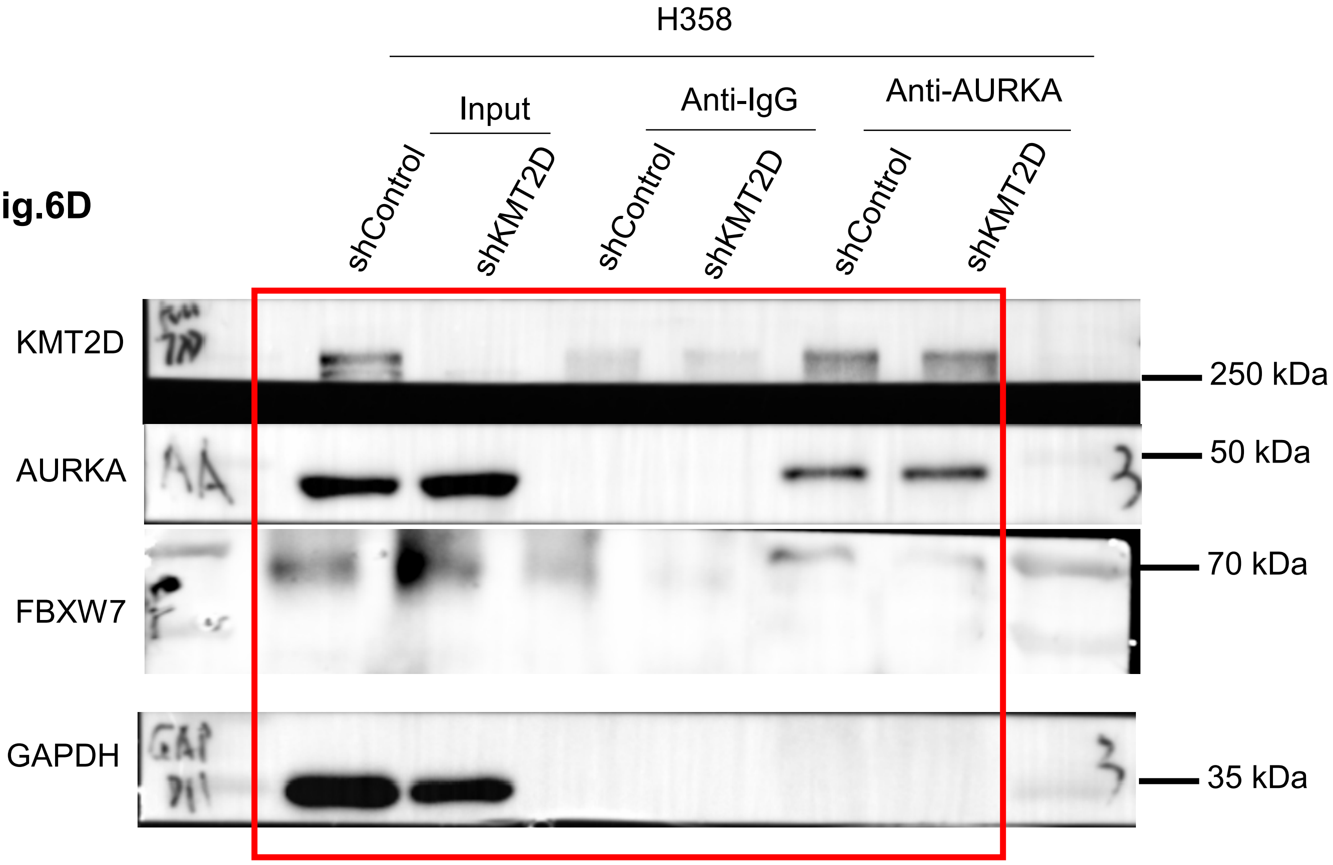

Fig.6E

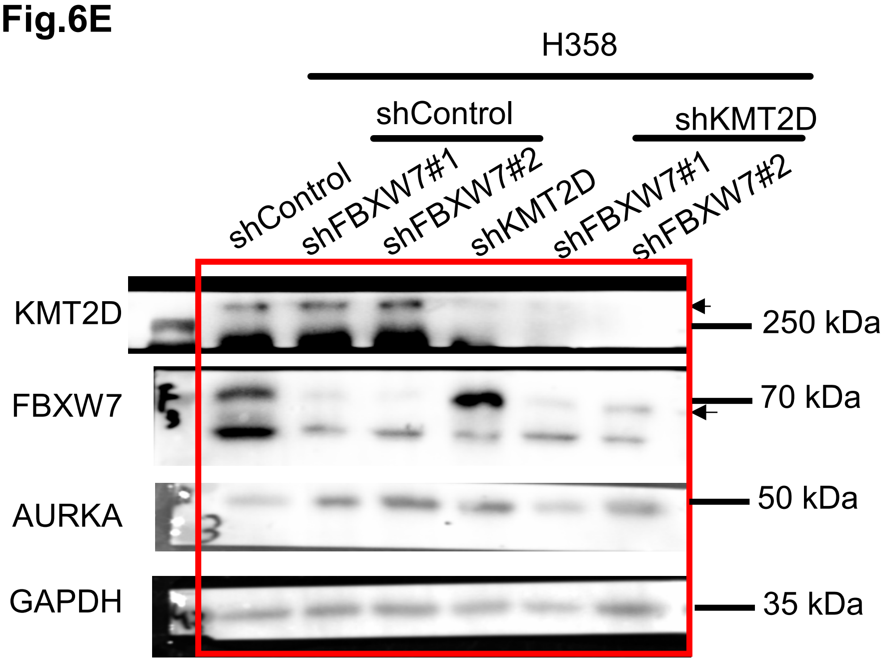
H358
shControl
shKMT2D
shControl
shFBXW7#1
shFBXW7#2
shKMT2D
shFBXW7#1
shFBXW7#2
KMT2D
FBXW7
AURKA
GAPDH
250 kDa
70 kDa
50 kDa
35 kDa

Fig.6F

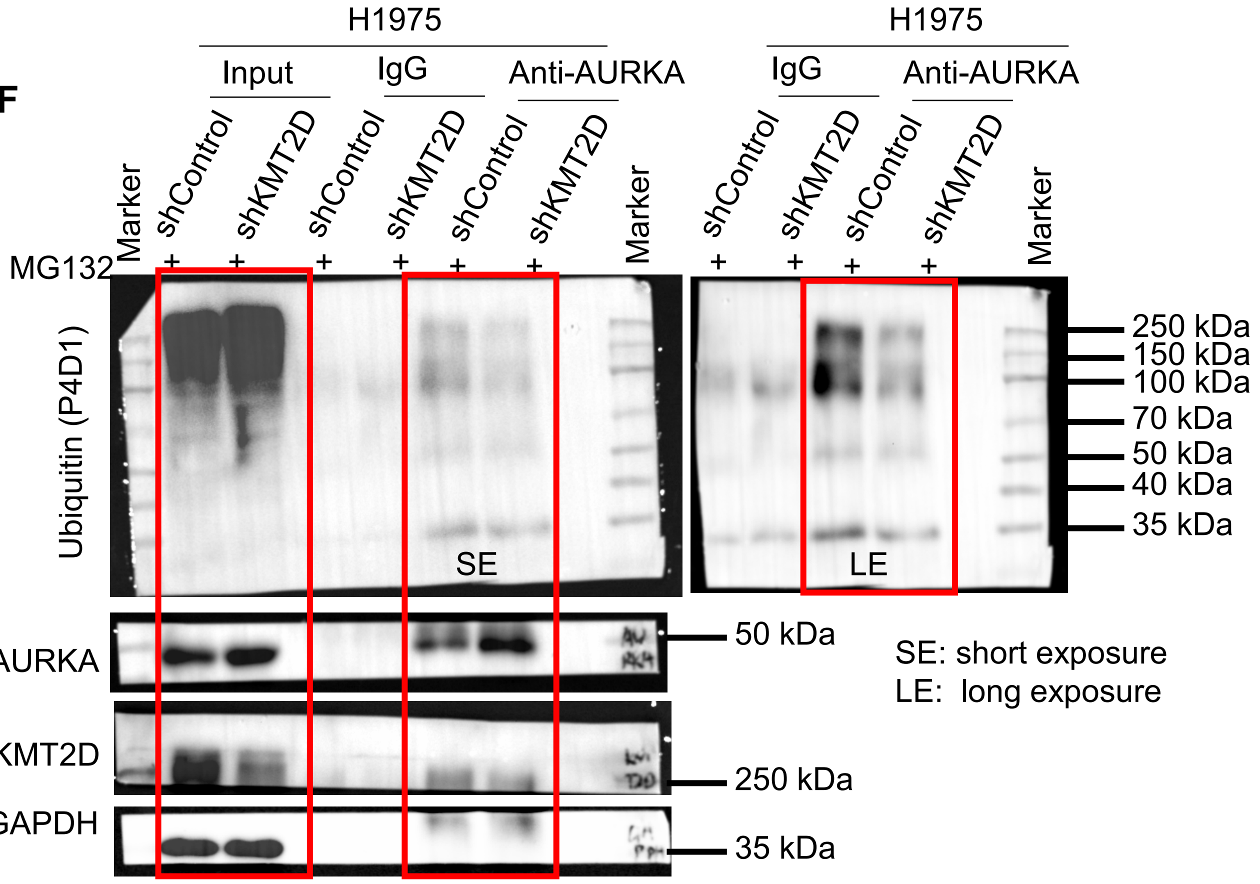
H1975
Input
IgG
Anti-AURKA
Marker
shControl
shKMT2D
shControl
shKMT2D
shControl
shKMT2D
Marker
H1975
IgG
Anti-AURKA
shControl
shKMT2D
shControl
shKMT2D
Marker
MG132
+
+
+
+
+
+
+
+
+
+
Ubiquitin (P4D1)
SE
LE
250 kDa
150 kDa
100 kDa
70 kDa
50 kDa
40 kDa
35 kDa
AURKA
KMT2D
GAPDH
50 kDa
250 kDa
35 kDa
SE: short exposure
LE: long exposure

Supplementary Fig.6E

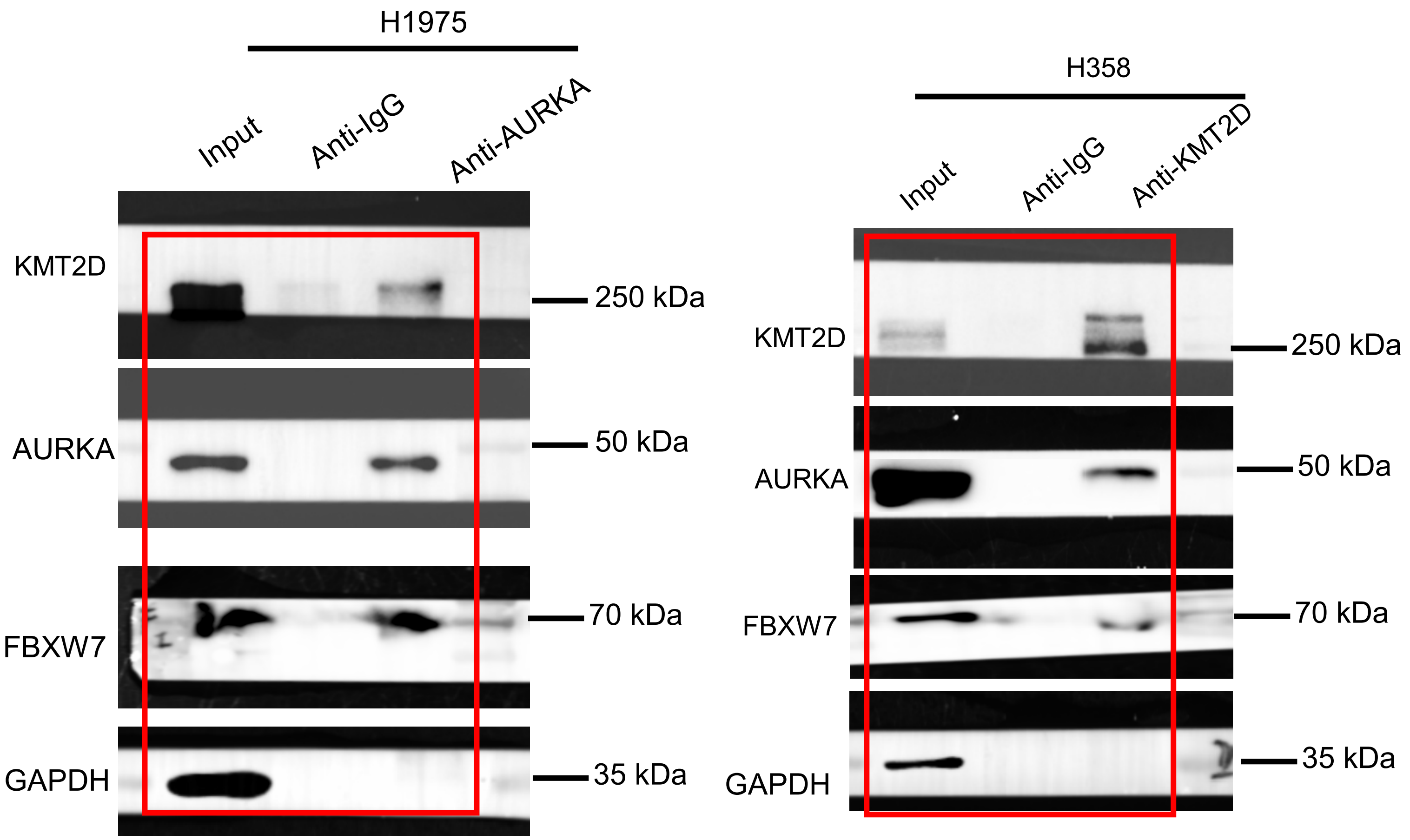

Supplementary Fig.6F

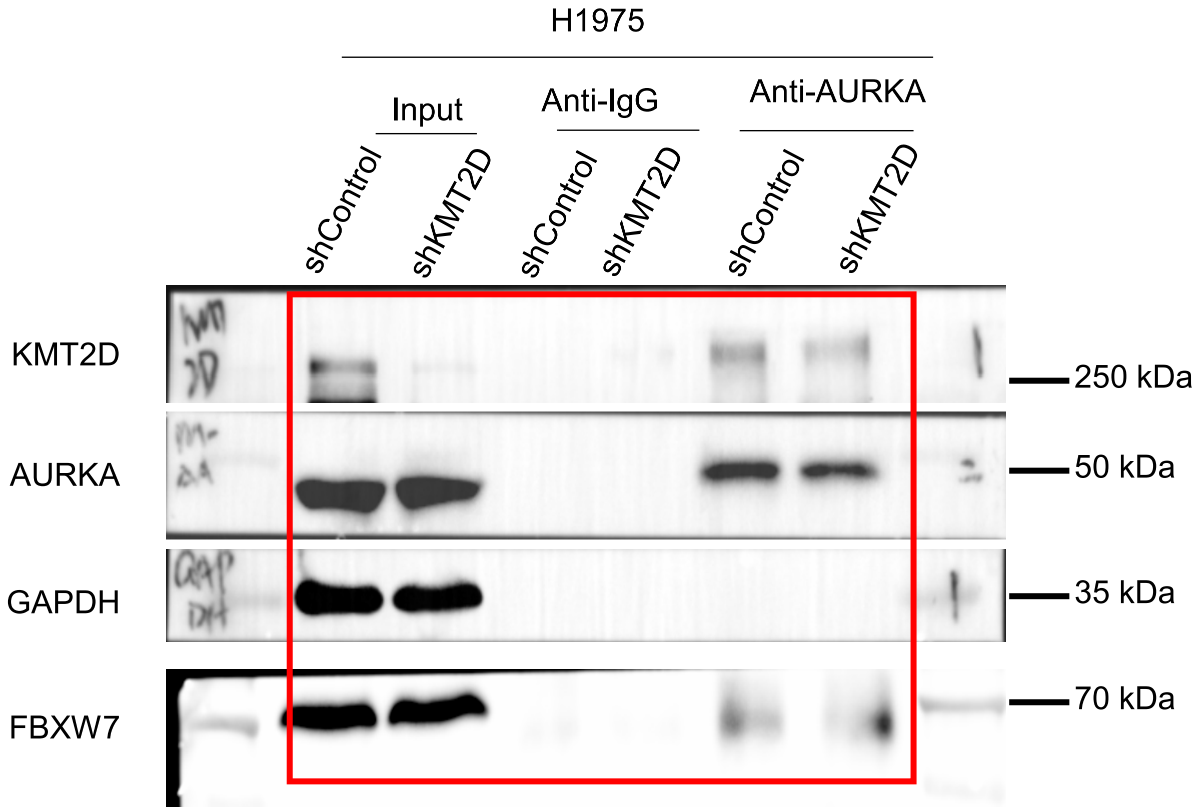

Fig.7A

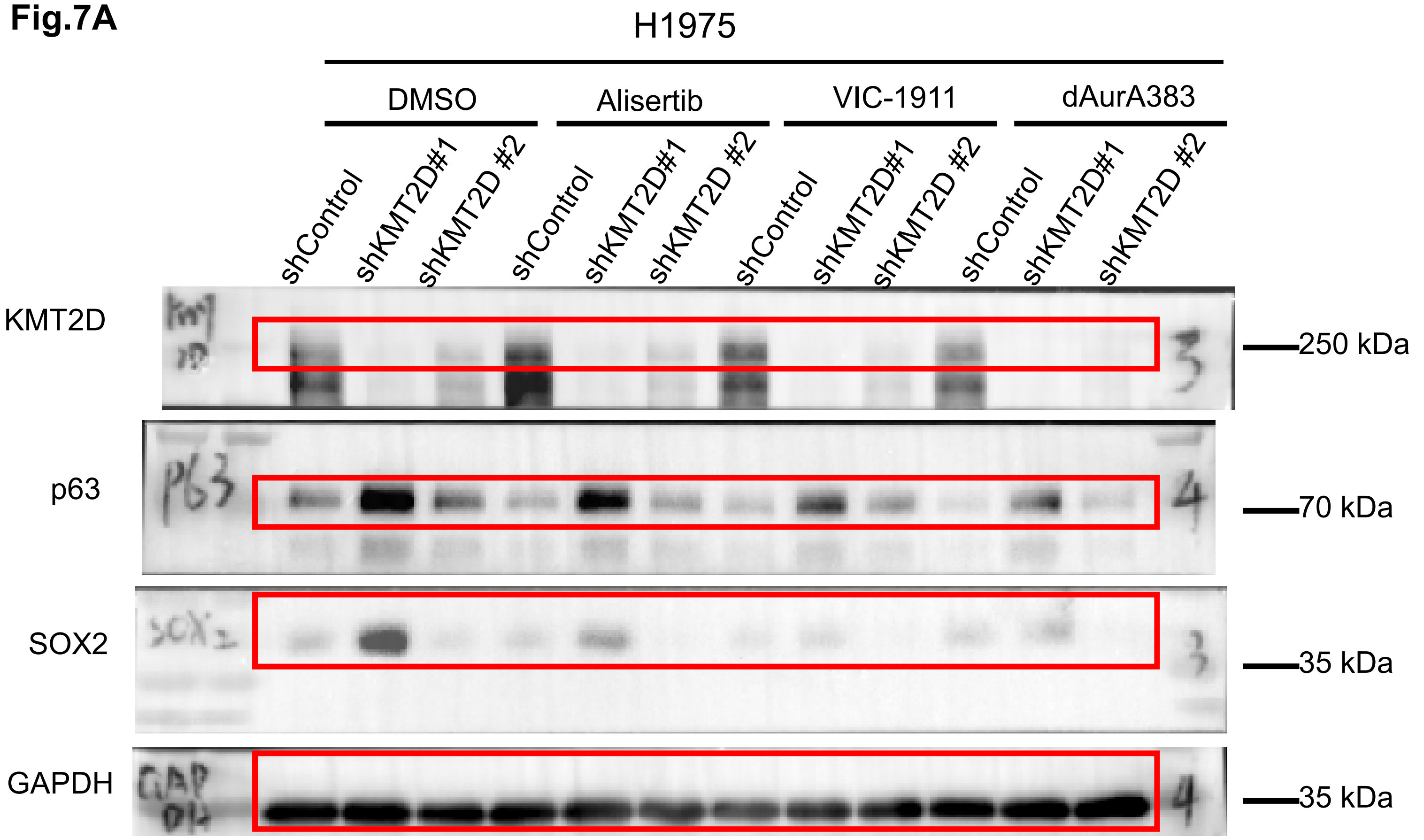

Supplementary Fig.7A

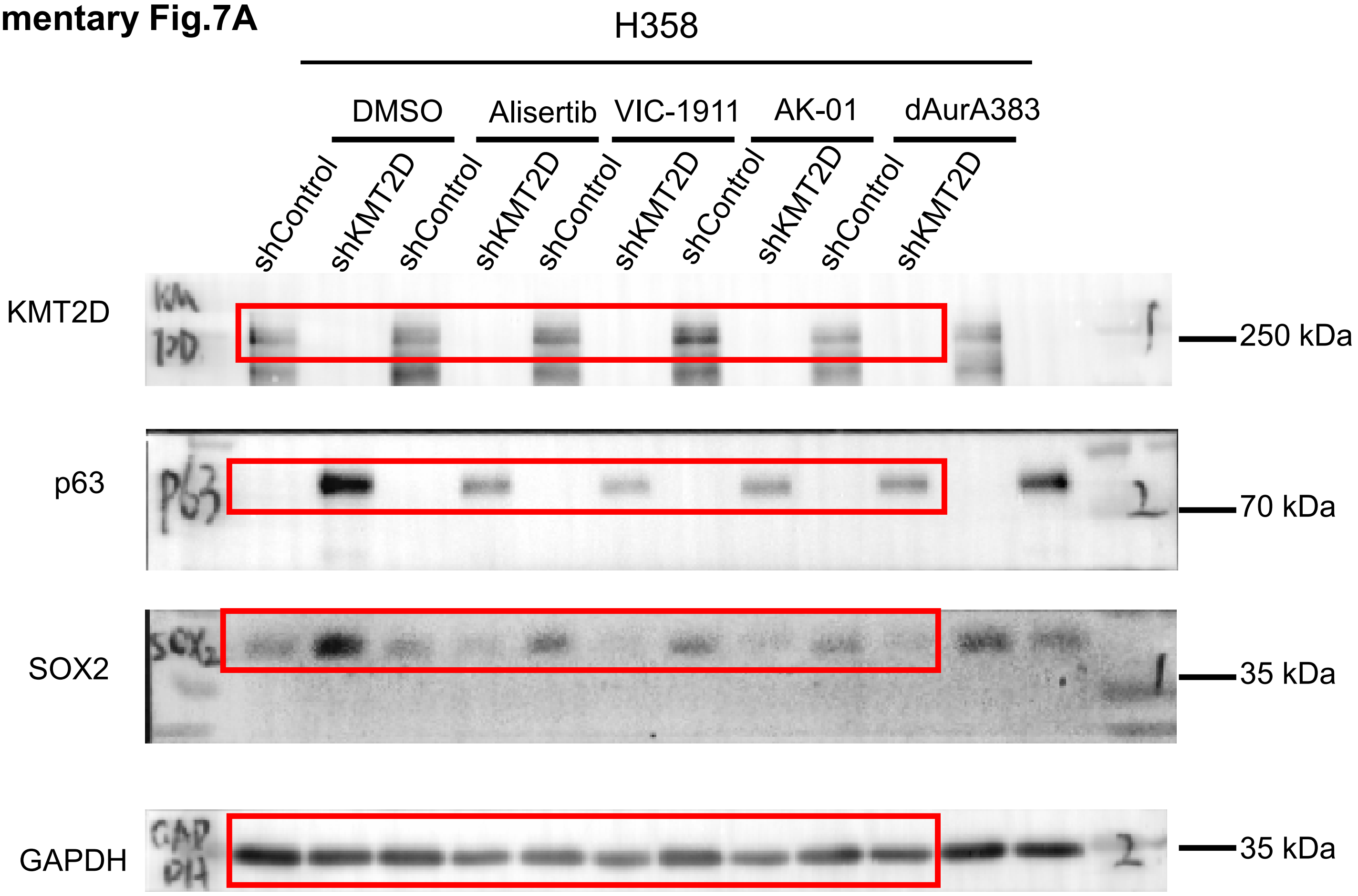

Supplementary Fig.7F

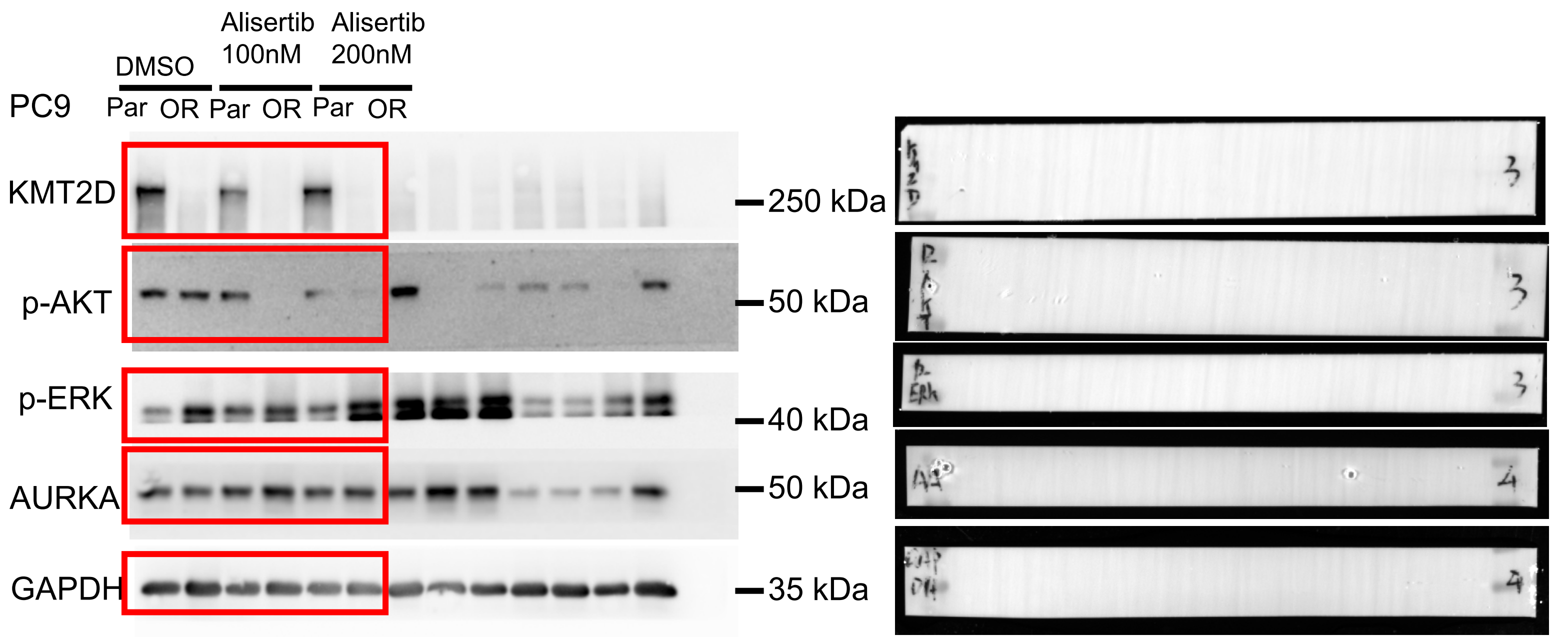

Supplement: Supplementary file 8 — CDD-25-3285RR_Original Data File 1 [file 41418_2025_1657_MOESM8_ESM.pdf]
